# Muscle-Strengthening and Conditioning Activities and Risk of Type 2 Diabetes: A Prospective Study in Two Cohorts of US Women

**DOI:** 10.1371/journal.pmed.1001587

**Published:** 2014-01-14

**Authors:** Anders Grøntved, An Pan, Rania A. Mekary, Meir Stampfer, Walter C. Willett, JoAnn E. Manson, Frank B. Hu

**Affiliations:** 1 Department of Nutrition, Harvard School of Public Health, Boston, Massachusetts, United States of America; 2 Department of Sport Science and Clinical Biomechanics, Research Unit for Exercise Epidemiology and Centre of Research in Childhood Health, University of Southern Denmark, Odense, Denmark; 3 Saw Swee Hock School of Public Health and Yong Loo Lin School of Medicine, National University of Singapore and National University Health System, Singapore, Republic of Singapore; 4 Department of Pharmaceutical Sciences, Massachusetts College of Pharmacy and Health Sciences University, Boston, Massachusetts, United States of America; 5 Department of Medicine, Harvard Medical School and Brigham and Women's Hospital, Boston, Massachusetts, United States of America; 6 Channing Division of Network Medicine, Department of Medicine, Harvard Medical School and Brigham and Women's Hospital, Boston, Massachusetts, United States of America; 7 Department of Epidemiology, Harvard School of Public Health, Boston, Massachusetts, United States of America; Lund University Diabetes Centre, Sweden

## Abstract

**Background:**

It is well established that aerobic physical activity can lower the risk of type 2 diabetes (T2D), but whether muscle-strengthening activities are beneficial for the prevention of T2D is unclear. This study examined the association of muscle-strengthening activities with the risk of T2D in women.

**Methods and Findings:**

We prospectively followed up 99,316 middle-aged and older women for 8 years from the Nurses' Health Study ([NHS] aged 53–81 years, 2000–2008) and Nurses' Health Study II ([NHSII] aged 36–55 years, 2001–2009), who were free of diabetes, cancer, and cardiovascular diseases at baseline. Participants reported weekly time spent on resistance exercise, lower intensity muscular conditioning exercises (yoga, stretching, toning), and aerobic moderate and vigorous physical activity (MVPA) at baseline and in 2004/2005. Cox regression with adjustment for major determinants for T2D was carried out to examine the influence of these types of activities on T2D risk. During 705,869 person years of follow-up, 3,491 incident T2D cases were documented. In multivariable adjusted models including aerobic MVPA, the pooled relative risk (RR) for T2D for women performing 1–29, 30–59, 60–150, and >150 min/week of total muscle-strengthening and conditioning activities was 0.83, 0.93, 0.75, and 0.60 compared to women reporting no muscle-strengthening and conditioning activities (*p*<0.001 for trend). Furthermore, resistance exercise and lower intensity muscular conditioning exercises were each independently associated with lower risk of T2D in pooled analyses. Women who engaged in at least 150 min/week of aerobic MVPA and at least 60 min/week of muscle-strengthening activities had substantial risk reduction compared with inactive women (pooled RR = 0.33 [95% CI 0.29–0.38]). Limitations to the study include that muscle-strengthening and conditioning activity and other types of physical activity were assessed by a self-administered questionnaire and that the study population consisted of registered nurses with mostly European ancestry.

**Conclusions:**

Our study suggests that engagement in muscle-strengthening and conditioning activities (resistance exercise, yoga, stretching, toning) is associated with a lower risk of T2D. Engagement in both aerobic MVPA and muscle-strengthening type activity is associated with a substantial reduction in the risk of T2D in middle-aged and older women.

*Please see later in the article for the Editors' Summary*

## Abstract 2

Anders Grøntved and colleagues examined whether women who perform muscle-strengthening and conditioning activities have an associated reduced risk of type 2 diabetes mellitus.

*Please see later in the article for the Editors' Summary*

## Editors' Summary

**Background:**

Worldwide, more than 370 million people have diabetes mellitus, a disorder characterized by poor glycemic control—dangerously high amounts of glucose (sugar) in the blood. Blood sugar levels are normally controlled by insulin, a hormone released by the pancreas. In people with type 2 diabetes (the commonest form of diabetes), blood sugar control fails because the fat and muscle cells that normally respond to insulin by removing excess sugar from the blood become less responsive to insulin. Type 2 diabetes, which was previously known as adult-onset diabetes, can often initially be controlled with diet and exercise, and with antidiabetic drugs such as metformin and sulfonylureas. However, as the disease progresses, the pancreatic beta cells, which make insulin, become impaired and patients may eventually need insulin injections. Long-term complications of diabetes, which include an increased risk of cardiovascular problems such as heart disease and stroke, reduce the life expectancy of people with diabetes by about 10 years compared to people without diabetes.

**Why Was This Study Done?:**

Type 2 diabetes is becoming increasingly common worldwide so better preventative strategies are essential. It is well-established that regular aerobic exercise—physical activity in which the breathing and heart rate increase noticeably such as jogging, brisk walking, and swimming—lowers the risk of type 2 diabetes. The World Health Organization currently recommends that adults should do at least 150 min/week of moderate-to-vigorous aerobic physical activity to reduce the risk of diabetes and other non-communicable diseases. It also recommends that adults should undertake muscle-strengthening and conditioning activities such as weight training and yoga on two or more days a week. However, although studies have shown that muscle-strengthening activity improves glycemic control in people who already have diabetes, it is unclear whether this form of exercise prevents diabetes. In this prospective cohort study (a study in which disease development is followed up over time in a group of people whose characteristics are recorded at baseline), the researchers investigated the association of muscle-strengthening activities with the risk of type 2 diabetes in women.

**What Did the Researchers Do and Find?:**

The researchers followed up nearly 100,000 women enrolled in the Nurses' Health Study (NHS) and the Nurses' Health Study II (NHSII), two prospective US investigations into risk factors for chronic diseases in women, for 8 years. The women provided information on weekly participation in muscle-strengthening exercise (for example, weight training), lower intensity muscle-conditioning exercises (for example, yoga and toning), and aerobic moderate and vigorous physical activity (aerobic MVPA) at baseline and 4 years later. During the study 3,491 women developed diabetes. After allowing for major risk factors for type 2 diabetes (for example, diet and a family history of diabetes) and for aerobic MVPA, compared to women who did no muscle-strengthening or conditioning exercise, the risk of developing type 2 diabetes among women declined with increasing participation in muscle-strengthening and conditioning activity. Notably, women who did more than 150 min/week of these types of exercise had 40% lower risk of developing diabetes as women who did not exercise in this way at all. Muscle-strengthening and muscle-conditioning exercise were both independently associated with reduced diabetes risk, and women who engaged in at least 150 min/week of aerobic MVPA and at least 60 min/week of muscle-strengthening exercise were a third as likely to develop diabetes as inactive women.

**What Do These Findings Mean?:**

These findings show that, among the women enrolled in NHS and NHSII, engagement in muscle-strengthening and conditioning activities lowered the risk of type 2 diabetes independent of aerobic MVPA. That is, non-aerobic exercise provided protection against diabetes in women who did no aerobic exercise. Importantly, they also show that doing both aerobic exercise and muscle-strengthening exercise substantially reduced the risk of type 2 diabetes. Because nearly all the participants in NHS and NHSII were of European ancestry, these results may not be generalizable to women of other ethnic backgrounds. Moreover, the accuracy of these findings may be limited by the use of self-administered questionnaires to determine how much exercise the women undertook. Nevertheless, these findings support the inclusion of muscle-strengthening and conditioning exercises in strategies designed to prevent type 2 diabetes in women, a conclusion that is consistent with current guidelines for physical activity among adults.

**Additional Information:**

Please access these websites via the online version of this summary at http://dx.doi.org/10.1371/journal.pmed.1001587.

- The US National Diabetes Information Clearinghouse provides information about diabetes for patients, health-care professionals and the general public, including information on diabetes prevention (in English and Spanish)
- The UK National Health Service Choices website provides information for patients and carers about type 2 diabetes and explains the benefits of regular physical activity
- The World Health Organization provides information about diabetes and about physical activity and health (in several languages); its 2010 Global Recommendations on Physical Activity for Health are available in several languages
- The US Centers for Disease Control and Prevention provides information on physical activity for different age groups; its Physical Activity for Everyone web pages include guidelines, instructional videos and personal success stories
- More information about the Nurses Health Study and the Nurses Health Study II is available
- The UK charity Healthtalkonline has interviews with people about their experiences of diabetes
- MedlinePlus provides links to further resources and advice about diabetes and about physical exercise and fitness (in English and Spanish)

## Introduction

The benefit of aerobic physical activity for preventing type 2 diabetes (T2D) is well established. Regular engagement in aerobic activities such as jogging, running, and brisk walking is associated with substantially lower risk of T2D [1],[2]. In the management and treatment of T2D, the importance of muscle-strengthening activity alone or in combination with aerobic exercise has received increasing attention [3]–[8]. While the cumulative evidence from randomized controlled trials show that glycemic control can be improved by muscle-strengthening activity among individuals with T2D [9], the evidence on the role of muscle-strengthening activity in T2D prevention is more limited. In a recent epidemiological study, we found that weight training was associated with a reduced risk of T2D independent of aerobic physical activity in men [10]. However, no such data are available among women.

Lower intensity muscular conditioning exercises such as yoga are popular physical activities among women. These types of activities may also be valuable for increasing and maintaining muscle strength and endurance [11],[12]; however, their role in T2D prevention is unknown. Furthermore, whether adding muscle-strengthening type activities to aerobic moderate and vigorous physical activity (MVPA), as endorsed in the current public health guidelines for physical activity among adults [13]–[15], provides additional benefit for T2D prevention is unclear.

The objective of this study was to examine the associations of muscle-strengthening and conditioning activities with the risk of T2D in two cohorts of US women followed prospectively in the Nurses' Health Study (NHS) and Nurses' Health Study II (NHSII). We also examined the independent and joint association of adherence to the current public health guidelines for participation in muscle-strengthening and conditioning activities and aerobic MVPA with T2D risk.

## Methods

### Ethics Statement

The Human Research Committee of Brigham and Women's Hospital (Boston, Massachusetts) and the institutional review board Harvard School of Public Health (Boston, Massachusetts) approved the study. Completion of the self-administered questionnaire was considered to imply informed consent.

### Study Population

The NHS and NHSII are two ongoing prospective cohort studies of female registered nurses. The NHS included 121,700 nurses aged 30 to 55 years at baseline in 1976 and the NHSII included 116,677 nurses aged 24 to 44 years at baseline in 1989. Questionnaires were sent to the participants every two years to update their information on disease status and major lifestyles, such as weight, smoking history, and physical activity. In the current analysis, we used the year 2000 for NHS and 2001 for NHS II as the baseline, because the information on muscle-strengthening and conditioning activities were first enquired in these years. We excluded women who reported a history of diabetes, cancer, myocardial infarction, angina, coronary artery bypass graft, other heart conditions, stroke, or pulmonary embolism on the baseline questionnaire (1976 or 1989) through 2000 or 2001 Furthermore, we excluded women who did not return information on physical activity and other important covariates for this analysis. This left us with study populations of 51,642 participants from NHS (aged 53–81 years at baseline in 2000) and 47,674 from NHSII (aged 36–55 at baseline in 2001) with information on T2D and relevant exposures and covariates.

### Assessment of Physical Activities

In 2000/2001 and 2004/2005 each participant reported her average weekly amount of resistance exercise, lower intensity exercise (yoga, stretching, toning), and aerobic physical activities. There were ten response categories ranging from none to >11 hours/week activities for these physical activities. Participants were also asked about their usual walking pace (easy <3.2 km/h, normal 3.2–4.6 km/h, brisk 4.7–6.5 km/h, very brisk >6.5 km/h). Aerobic physical activities included brisk walking (for exercise or to work), jogging, running, bicycling, tennis, swimming, other aerobic exercise (aerobic, dance, ski or stair machine, etc.), daily number of flights of stairs climbed, and other vigorous activities. We considered these aerobic activities of at least moderate intensity (≥3 metabolic equivalent of tasks [METs]) because these activities are usually performed repetitively and produce dynamic contractions of large muscle groups for an extended period of time [14]. To represent the public health recommendation to undertake muscle-strengthening type activity, we calculated the total time spent on muscle-strengthening and conditioning activity (sum of resistance exercise and lower intensity exercise like yoga, stretching, and toning). Examples of muscle-strengthening activity include resistance exercise with free weights, weight machines, exercises against own weight, yoga, and outdoor work [14]. It is currently recommended that adults should engage in at least 150 min/week of aerobic activity of at least moderate intensity and do muscle-strengthening activity two or more days/week [13]–[15]. For each type of activity (aerobic- and muscle-strengthening activity), we grouped participants into five categories: none, 1–29 min/week, 30–59 min/week, 60–50 min/week, and more than 150 min/week. We have previously documented the validity of the physical activity questionnaire in a sub-sample of the NHSII participants. The Pearson correlation between MVPA as reported in diaries and that reported on the questionnaires was 0.62 [16]. We did not evaluate the validity for resistance exercise or lower intensity conditioning exercise; however, in a sample of men with fairly similar age-range from Health Professional Follow-up Study (HPFS) using a similar question for weight training, the de-attenuated correlation was 0.79 with the average of four weekly diaries across different seasons and that reported in the 1992 questionnaire [17].

### Assessment of Type 2 Diabetes

Women who reported a diagnosis of diabetes in the biennial follow-up questionnaires were sent a supplementary questionnaire to confirm the diagnosis, obtaining information on symptoms, treatment, and diagnostic tests. We used the American Diabetes Association criteria [18] to confirm self-reported diagnosis of T2D: (1) one or more classic symptoms (excessive thirst, polyuria, weight loss, hunger) plus fasting plasma glucose concentrations of at least 7.0 mmol/l or random plasma glucose concentrations of at least 11.1 mmol/l; (2) at least two elevated plasma glucose concentrations on different occasions (fasting concentrations of at least 7.0 mmol/l, random plasma glucose concentrations of at least 11.1 mmol/l, and/or concentrations of at least 11.1 mmol/l after ≥2 h shown by oral glucose tolerance testing) in the absence of symptoms; or (3) treatment with hypoglycemic medication (insulin or oral hypoglycemic agent). In a validation study in a subgroup of NHS participants, 98% (61 of 62) of self-reported T2D cases were confirmed by means of medical record review in a validation study in a sub-group of NHS participants [19]. In another sub-study that assessed the prevalence of undiagnosed T2D in NHS, fasting plasma glucose and plasma fructosamine were measured in a random sample of participants (n = 200) who did not report a previous diagnosis of T2D [20]. One (0.5%) of the women had an elevated fasting plasma glucose or plasma fructosamine level in the diabetic range.

### Assessment of Covariates

We obtained family history of T2D based on questionnaires completed in 1982, 1988, and 1992 among women from the NHS and in 1989, 1997, and 2001 among women from the NHSII. A history of hypertension and high cholesterol were obtained biennially from 1976 through 2006 in the NHS and from 1989 through 2007 in the NHS II. Smoking status, body mass index (BMI), menopausal status, oral contraceptive use (NHSII only), and uses of post menopausal hormone therapy use were assessed at baseline and biennially during follow-up. Dietary factors were assessed in 1998 and 2002 for NHS participants and in 1999 and 2003 for NHSII participants using a 131-item validated food frequency questionnaire (FFQ) [21]. From these questionnaires we calculated cumulative average daily intake of trans fat (percent of total energy), total energy (cal/d), polyunsaturated fat to saturated fat ratio, cereal fiber (g/d), whole grains (g/d), glycemic load, alcohol (g/d), and coffee (cups/d). We also calculated a dietary index composed of polyunsaturated fat to saturated fat ratio, trans fat (inverted), cereal fiber, whole grains, and glycemic load (inverted) by standardizing and summarizing the respective continuously scaled dietary variables [22]. Race was determined by asking participants to indicate their major ancestry. Participants who identified themselves as Southern European, Scandinavian, and other Caucasian were assigned as white, and participants who identified themselves as Black, Hispanic, Asian, Native American, and other ancestry were assigned as non-white.

### Statistical Analysis

We calculated person-time at risk from the return of the 2000 (NHS) or 2001 (NHSII) questionnaire until June 30th 2008 (NHS) or June 30th 2009 (NHSII), death or loss to follow-up, whichever occurred first. Relative risks (RRs) of T2D by categories of total muscle-strengthening and conditioning activities, resistance exercise, lower intensity muscular conditioning exercise, and aerobic MVPA were estimated using time dependent Cox proportional-hazard regression. We stratified the analyses jointly by age (in months) at start of follow-up and the year of questionnaire return to control for calendar time and age and any possible two-way interactions between these two time scales. To best represent long-term exposure to activity we updated activity (e.g., muscle-strengthening activity) during follow-up by calculating the cumulative average. In multivariable analysis we additionally adjusted for race (white, non-white) and the biennially (or every four years) updated variables: alcohol intake (0, 1–5, >5 g/d), coffee intake (0, <1, 1–3, 3–5, >5 cups/day), smoking (never, past, or current), post menopausal hormone use (never, past, current), oral contraceptive use (only NHSII: never, past, current), menopausal status (only NHSII: pre, post), family history of diabetes (yes, no), total calorie intake, saturated fat to polyunsaturated fat ratio, trans fat, cereal fiber, whole grains, and glycemic load (all dietary factors in quintiles). We also assessed the extent to which the association of muscle-strengthening and conditioning activities with T2D risk was explained by adiposity by additionally adjusting for BMI (continuous [updated biennially]) and history of hypertension and raised cholesterol (yes, no [updated biennially]). Test for trend (Wald test) was examined by including the median activity time value in each category of activity (e.g., resistance exercise) and treated this as a continuous variable in the model. To explore the possibility of heterogeneous estimates of association by the level of BMI, a stratified analysis by categories of BMI were carried out (<25, 25 to <30, ≥30 kg/m^2^). We also examined whether the association differed by age (<65 and ≥65 years), family history of T2D, diet quality score, race (white/non-white), and aerobic MVPA (quintiles). Estimates of association from each cohort were pooled using a fixed effect model (weighted by the inverse of the variance). The two NHS cohorts are homogeneous across various study characteristics. There was no indication that the proportional hazards assumption was violated based on the assessment of interaction between follow-up time and muscle-strengthening or aerobic activities. The linearity of the relationship between type of activity (e.g., total muscle-strengthening activity) and T2D risk was evaluated by using restricted cubic spline regression with three or four knots, depending on the number of observations with non zero values of activity [23]. The knots were placed with equally spaced quantiles between them and the analyses were restricted to observations below activity time close to the 99th percentile. The estimates from these models were plotted and linearity was examined graphically and by comparing models with the cubic spline terms and models including only the linear term using the likelihood ratio test.

We then analyzed the independent and joint association of adherence to the current recommendations [13]–[15] of aerobic MVPA (≥150 min/week) and resistance exercise (at least two times/week, which we approximated to be ≥60 min/week)) with the risk of T2D.

Three types of sensitivity analyses were carried out. Firstly, we performed an analysis restricted to individuals reporting no aerobic MVPA when analyzing muscle-strengthening and conditioning activity to limit the possibility of residual confounding by other aerobic activity. Secondly, we carried out an analysis using a 2-year lag between exposure and T2D incidence to limit the possibility that subclinical T2D could influence participation in muscle-strengthening and conditioning activities. Thirdly, we repeated the analyses using the simple updated activity time (the recent time spent on activity) instead of the cumulative average.

All analyses were conducted in SAS version 9.2 (SAS Institute, Inc.).

## Results

We documented 2,158 and 1,333 new cases of T2D during 345,752 and 360,117 person years of follow-up in the NHS and NHSII, respectively. Table 1 shows the baseline characteristics of the study populations by levels of total muscle-strengthening and conditioning activities per week. Muscle-strengthening and conditioning activities at baseline were inversely associated with BMI, intake of transfat, and positively associated with aerobic MVPA, intake of alcohol, cereal fiber, whole grains, total energy intake, polyunsaturated fat to saturated fat ratio, and glycemic load in both cohorts of women. Furthermore, women who reported >150 min/week of muscle-strengthening and conditioning activities were less likely to smoke and to have a family history of T2D compared to women reporting no muscle-strengthening and conditioning activities. A total of 19% and 33% of women reported any resistance exercise at baseline in the NHS and NHSII, respectively. In addition 28% and 35% of women reported any lower intensity muscle conditioning exercise (yoga, stretching, toning) at baseline in the NHS and NHSII, respectively. Resistance exercise, lower intensity conditioning exercise, and aerobic MVPA were modestly correlated (Spearman's r: NHS r = 0.17–0.31, NHSII r = 0.31–0.42).

**Table 1:** Age-adjusted baseline characteristics of 51,642 participating women from the Nurses' Health Study and 47,674 women from the Nurses' Health Study II by minutes of muscle-strengthening and conditioning activity per week.

Characteristics	Minutes Per Week of Muscle-Strengthening and Conditioning Activity				
	None	1–29	30–59	60–150	>150
**Nurses' Health Study**					
*n*	32,908	5,138	4,766	6,036	2,794
Age (years)	65.1(7.0)	65.0(7.1)	65.1(7.0)	64.8(6.8)	64.4(6.7)
BMI (kg/m^2^)	26.8(5.2)	26.1(4.8)	25.9(4.8)	25.3(4.5)	24.8(4.3)
Aerobic MVPA^a^ (h/week)	1.8(2.8)	1.9(2.7)	2.6(3.1)	3.3(3.4)	4.6(4.2)
Alcohol intake (grams/day)	5.1(9.4)	5.3(9.0)	5.7(9.3)	6.1(9.1)	6.5(9.8)
Coffee intake (cups/day)	1.3(1.4)	1.2(1.4)	1.3(1.4)	1.2(1.3)	1.2(1.3)
P∶S ratio	0.6(0.2)	0.6(0.2)	0.6(0.2)	0.7(0.2)	0.7(0.3)
Transfat (% of total energy)	1.7(0.6)	1.6(0.6)	1.5(0.5)	1.4(0.5)	1.3(0.5)
Cereal fiber (grams/day)	6.5(3.7)	6.9(3.8)	7.2(3.7)	7.4(4.2)	7.5(4.0)
Whole grains (grams/day)	26.4(19.7)	29.0(20.0)	30.8(21.2)	32.1(21.7)	33.6(22.3)
Glycemic load	124(46)	127(45)	129(45)	129(45)	130(47)
Total energy intake (kcal/day)	1687(545)	1724(536)	1762(547)	1750(535)	1773(549)
Current smoking, %	11	7	8	6	5
Race, % white	98	97	98	97	98
Family history of diabetes, %	26	27	26	25	25
Post menopausal hormone use, %	50	52	52	57	56
History of hypertension, %	45	41	42	39	39
History of high cholesterol, %	58	58	57	55	54
**Nurses' Health Study II**					
*n*	24,978	6,719	4,512	7,060	4,405
Age (years)	46.2(4.7)	46.0(4.7)	46.3(4.7)	46.0(4.7)	46.0(4.6)
BMI (kg/m^2^)	27.5(6.5)	26.1(5.6)	25.8(5.5)	25.0(4.9)	24.5(4.7)
Aerobic MVPA^a^ (h/week)	1.6(2.7)	2.1(2.7)	3.0(3.2)	3.9(3.6)	6.2(5.6)
Alcohol intake (grams/day)	3.6(7.3)	4.2(7.4)	4.3(7.2)	4.9(7.5)	5.3(8.4)
Coffee intake (cups/day)	1.1(1.4)	1.1(1.4)	1.1(1.4)	1.1(1.4)	1.1(1.3)
P∶S ratio	0.6(0.2)	0.6(0.2)	0.6(0.2)	0.6(0.2)	0.6(0.3)
Transfat (% of total energy)	2.1(0.7)	1.9(0.7)	1.8(0.7)	1.7(0.7)	1.6(0.7)
Cereal fiber (grams/day)	6.4(3.5)	6.9(3.8)	7.2(3.8)	7.3(3.9)	7.3(3.9)
Whole grains (grams/day)	26.6(19.2)	30.2(20.9)	31.5(20.8)	32.4(21.9)	33.9(22.3)
Glycemic load	126(49)	128(48)	132(49)	129(48)	130(48)
Total energy intake (kcal/day)	1795(551)	1826(545)	1868(556)	1828(538)	1849(553)
Current smoking, %	9	7	8	6	8
Race, % white	97	96	97	97	97
Family history of diabetes, %	36	35	34	34	34
Menopausal status, %	31	30	31	31	32
Post menopausal hormone use among post menopausal women, %	44	44	45	46	46
Oral contraceptive use, %	87	86	88	88	88
History of hypertension, %	19	18	17	16	15
History of high cholesterol, %	14	11	12	10	9

Muscle-strengthening and conditioning activity is defined as resistance exercise and lower intensity conditioning exercise (yoga, stretching, toning). Values are means (standard deviation [SD]) or percentages and are standardized to the age distribution of the study population.

^a^ Aerobic MVPA consists of brisk walking, jogging, running, bicycling, tennis, swimming, other aerobic exercise, other vigorous exercise, and stair climbing.

P∶S ratio, polyunsaturated fat to saturated fat ratio.

**Figure 1:**
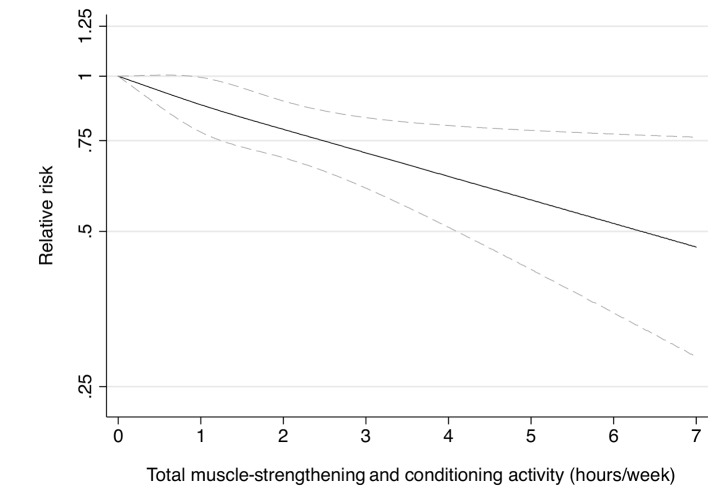
Dose-response relationship between total muscle-strengthening activity (hours/week) and risk of type 2 diabetes in women from the Nurses' Health Study. Dotted lines are 95% CI for the trend obtained from restricted cubic spline regression (3 knots) truncated at 7 hours/week (≈99th percentile). Estimates were adjusted for age (months), smoking (never, past, or current), alcohol consumption (0, 1–5, >5 g/d), coffee intake (0, <1, 1–3, 3–5, >5 cups/day), race (white, non-white), family history of diabetes, post menopausal hormone use (never, past, current), intake of total energy, trans fat, polyunsaturated fat to saturated fat ratio, cereal fiber, wholegrain, and glycemic load (all dietary factors in quintiles), aerobic physical activity (0, 1–29, 30–59, 60–150, >150 min/week). *p* = 0.82 for non-linear response.

**Figure 2:**
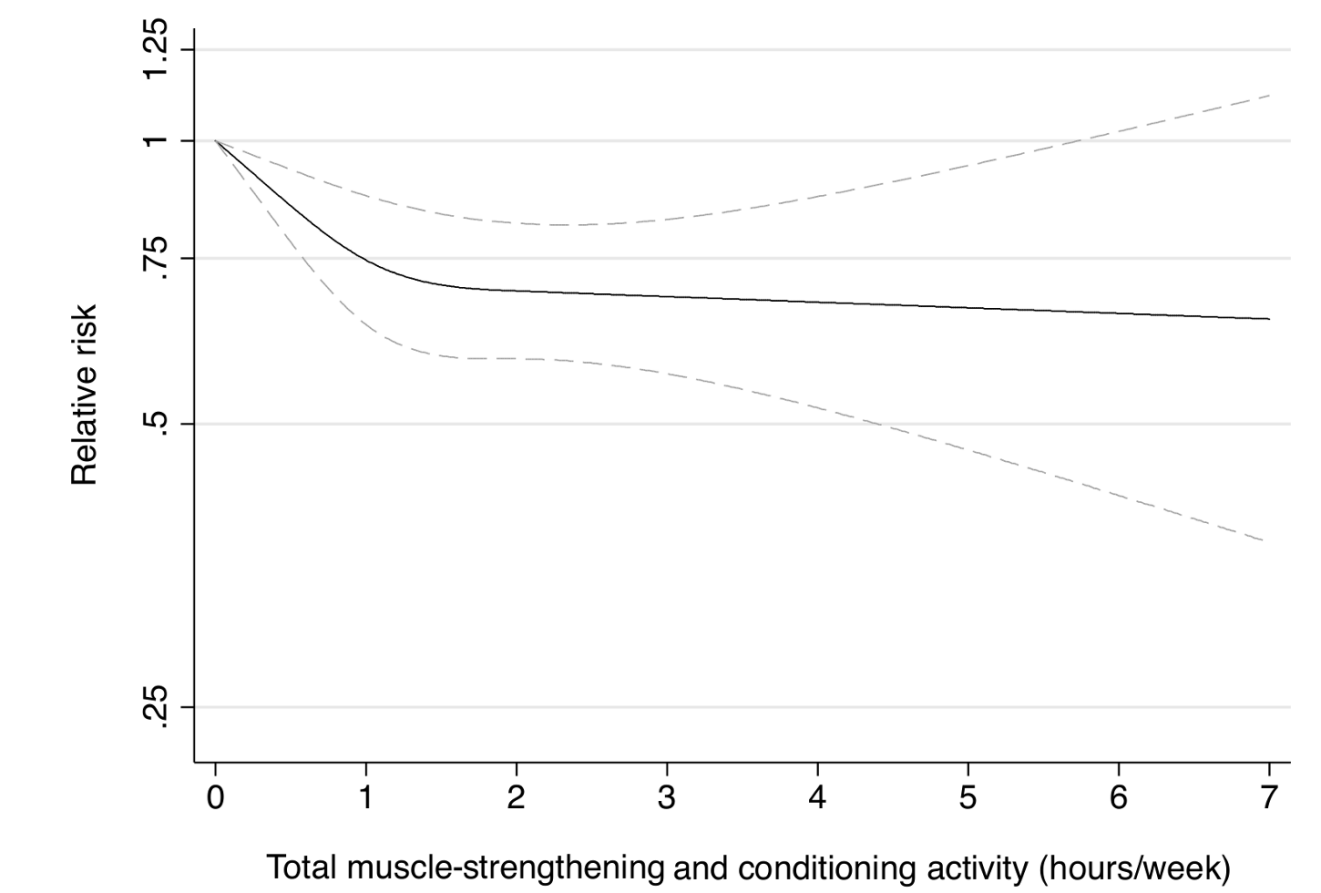
Dose-response relationship between total muscle-strengthening activity (hours/week) and risk of type 2 diabetes in women from the Nurses' Health Study II. Dotted lines are 95% CI for the trend obtained from restricted cubic spline regression (3 knots) truncated at 7 hours/week (≈99th percentile). Estimates were adjusted for age (months), smoking (never, past, or current), alcohol consumption (0, 1–5, >5 g/d), coffee intake (0, <1, 1–3, 3–5, >5 cups/day), race (white, non-white), family history of diabetes, post menopausal hormone use (never, past, current), intake of total energy, trans fat, polyunsaturated fat to saturated fat ratio, cereal fiber, wholegrain, and glycemic load (all dietary factors in quintiles), oral contraceptive use (never, past, current), menopausal status (pre, post), and aerobic physical activity (0, 1–29, 30–59, 60–150, >150 min/week). *p* = 0.02 for non-linear response.

**Figure 3:**
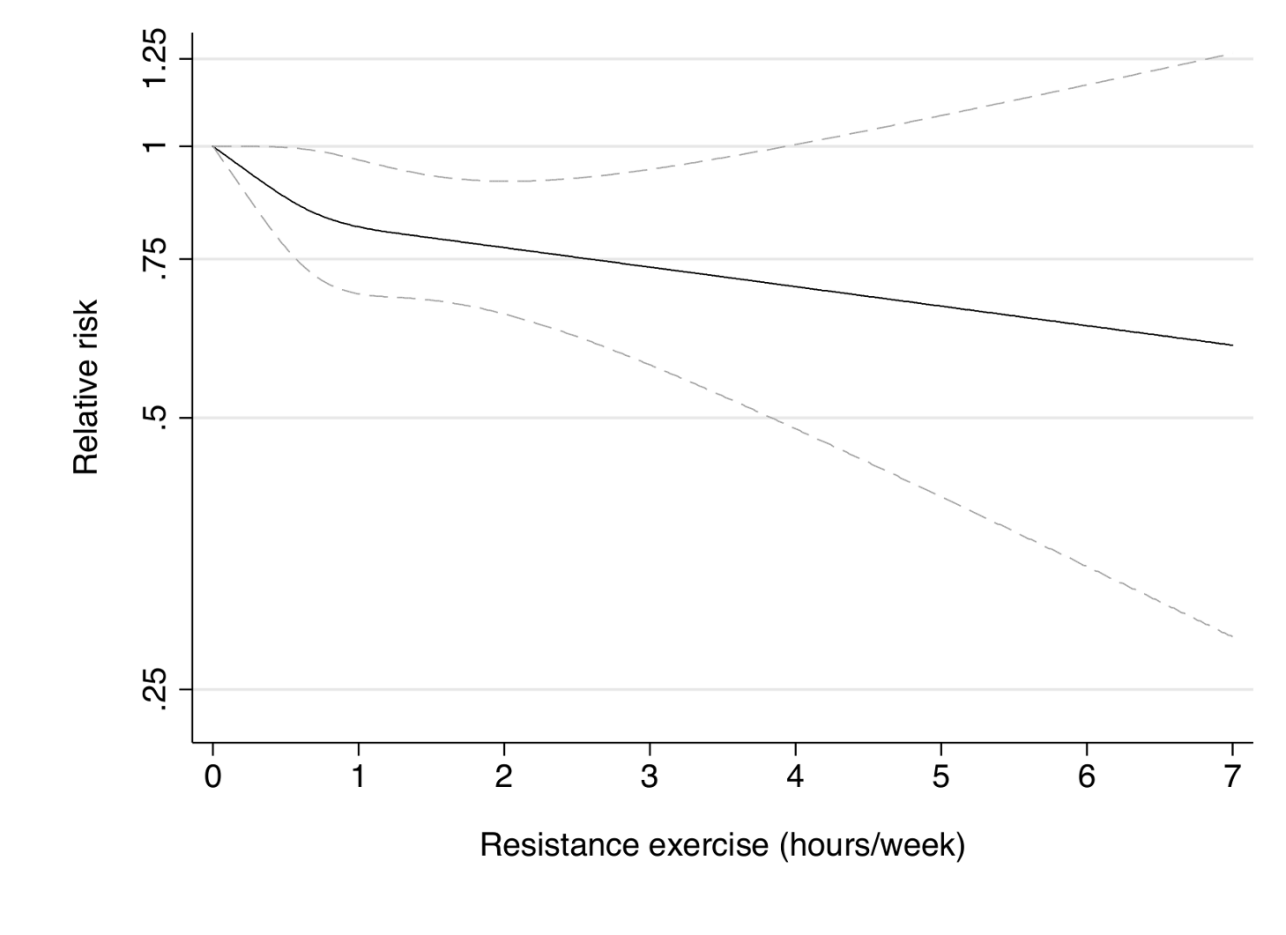
Dose-response relationship between resistance exercise (hours/week) and risk of type 2 diabetes in women from the Nurses' Health Study. Dotted lines are 95% CI for the trend obtained from restricted cubic spline regression (3 knots) truncated at 7 hours/week (≈99th percentile). Estimates were adjusted for age (months), smoking (never, past, or current), alcohol consumption (0, 1–5, >5 g/d), coffee intake (0, <1, 1–3, 3–5, >5 cups/day), race (white, non-white), family history of diabetes, post menopausal hormone use (never, past, current), intake of total energy, trans fat, polyunsaturated fat to saturated fat ratio, cereal fiber, wholegrain, and glycemic load (all dietary factors in quintiles), aerobic physical activity (0, 1–29, 30–59, 60–150, >150 min/week), and lower intensity muscular conditioning exercises (0, 1–29, 30–59, 60–150, >150 min/week). *p* = 0.24 for non-linear response.

**Figure 4:**
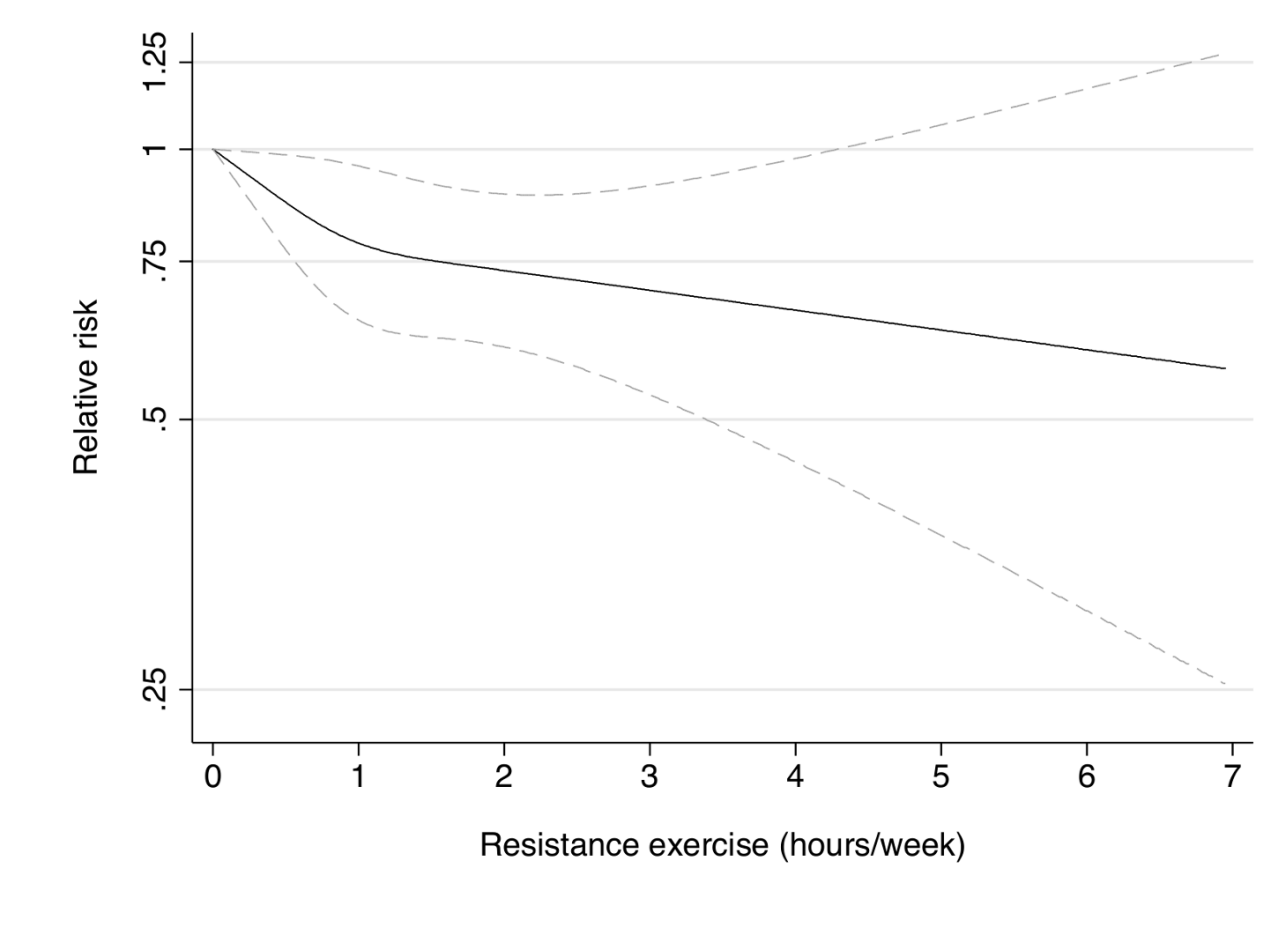
Dose-response relationship between resistance exercise (hours/week) and risk of type 2 diabetes in women from the Nurses' Health Study II. Dotted lines are 95% CI for the trend obtained from restricted cubic spline regression (3 knots) truncated at 7 hours/week (≈99th percentile). Estimates were adjusted for age (months), smoking (never, past, or current), alcohol consumption (0, 1–5, >5 g/d), coffee intake (0, <1, 1–3, 3–5, >5 cups/day), race (white, non-white), family history of diabetes, post menopausal hormone use (never, past, current), intake of total energy, trans fat, polyunsaturated fat to saturated fat ratio, cereal fiber, wholegrain, and glycemic load (all dietary factors in quintiles), oral contraceptive use (never, past, current), menopausal status (pre, post), and aerobic physical activity (0, 1–29, 30–59, 60–150, >150 min/week), and lower intensity muscular conditioning exercises (0, 1–29, 30–59, 60–150, >150 min/week). *p* = 0.22 for non-linear response.

**Figure 5:**
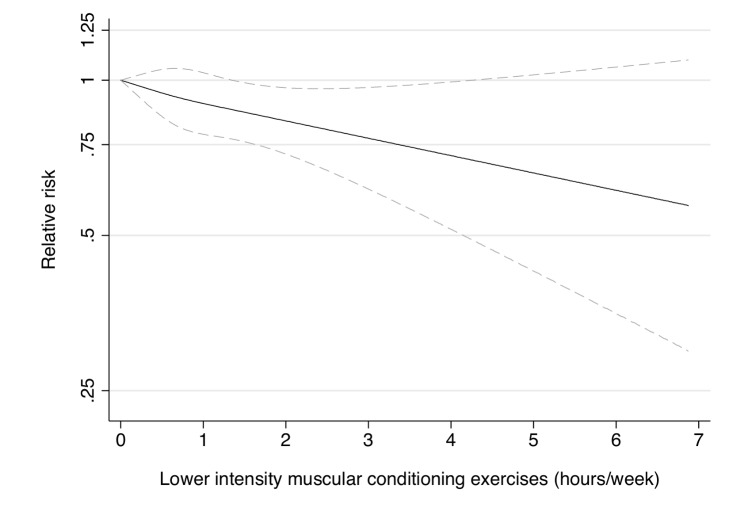
Dose-response relationship between lower intensity muscular conditioning exercises (hours/week) and risk of type 2 diabetes in women from the Nurses' Health Study. Dotted lines are 95% CI for the trend obtained from restricted cubic spline regression (3 knots) truncated at 7 hours/week (≈99th percentile). Estimates were adjusted for age (months), smoking (never, past, or current), alcohol consumption (0, 1–5, >5 g/d), coffee intake (0, <1, 1–3, 3–5, >5 cups/day), race (white, non-white), family history of diabetes, post menopausal hormone use (never, past, current), intake of total energy, trans fat, polyunsaturated fat to saturated fat ratio, cereal fiber, wholegrain, and glycemic load (all dietary factors in quintiles), aerobic physical activity (0, 1–29, 30–59, 60–150, >150 min/week), and resistance exercise (0, 1–29, 30–59, 60–150, >150 min/week). *p* = 0.80 for non-linear response.

**Figure 6:**
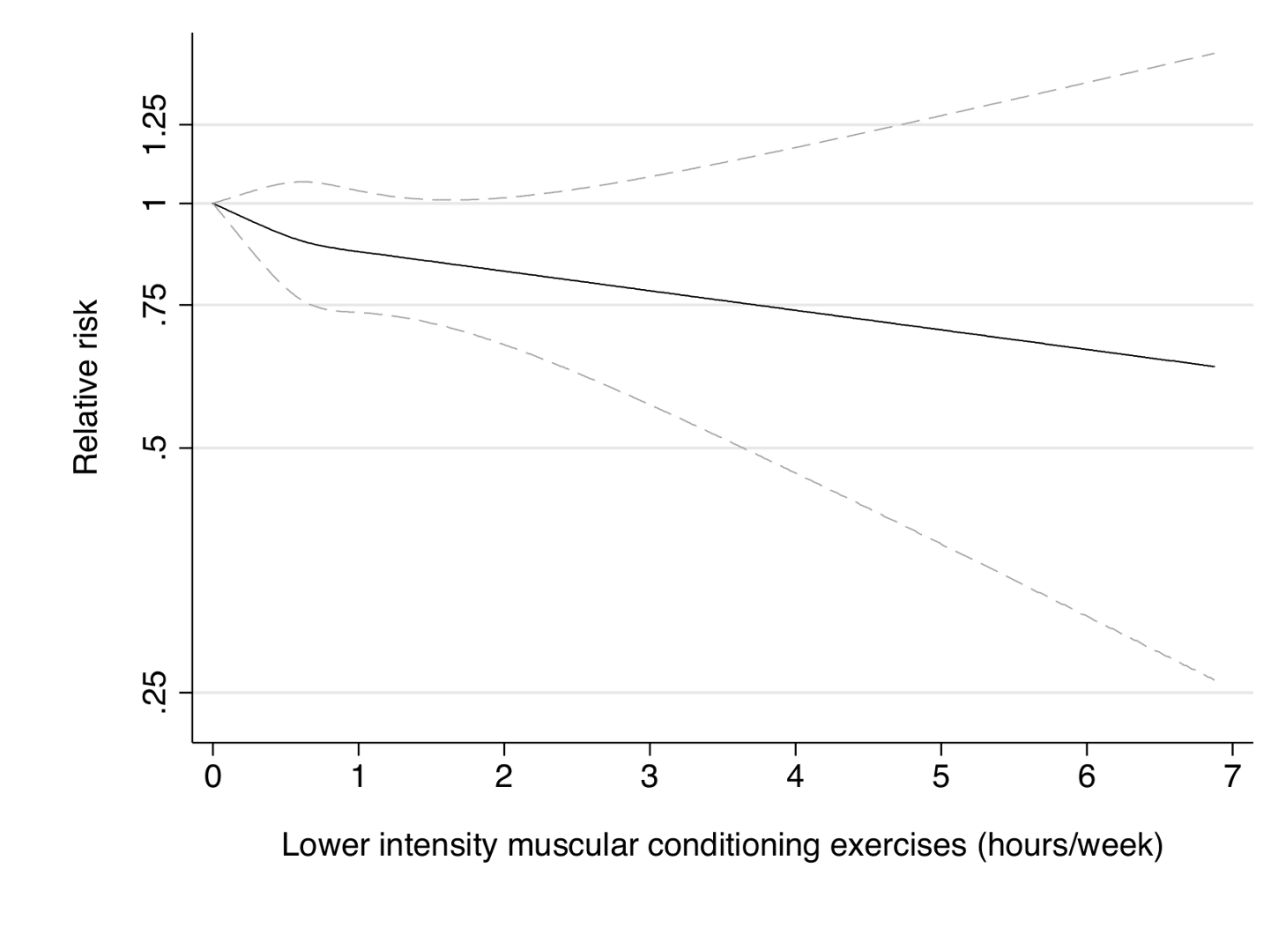
Dose-response relationship between resistance exercise (hours/week) and risk of type 2 diabetes in women from the Nurses' Health Study II. Dotted lines are 95% CI for the trend obtained from restricted cubic spline regression (3 knots) truncated at 7 hours/week (≈99th percentile). Estimates were adjusted for age (months), smoking (never, past, or current), alcohol consumption (0, 1–5, >5 g/d), coffee intake (0, <1, 1–3, 3–5, >5 cups/day), race (white, non-white), family history of diabetes, post menopausal hormone use (never, past, current), intake of total energy, trans fat, polyunsaturated fat to saturated fat ratio, cereal fiber, wholegrain, and glycemic load (all dietary factors in quintiles), oral contraceptive use (never, past, current), menopausal status (pre, post), and aerobic physical activity (0, 1–29, 30–59, 60–150, >150 min/week), and resistance exercise (0, 1–29, 30–59, 60–150, >150 min/week). *p* = 0.53 for non-linear response.

Participation in muscle-strengthening and conditioning activities was associated with a decreased risk of T2D in multivariable-adjusted analysis, with and without adjustment for aerobic MVPA, in both cohorts of women (Table 2). The pooled RR for T2D for women performing 1–29, 30–59, 60–150, and >150 min/week of muscle-strengthening and conditioning activities was 0.83, 0.93, 0.75, and 0.60 compared with women reporting no muscle-strengthening and conditioning activities (*p*<0.001 for trend). When analyzed separately, both resistance exercise and lower intensity muscular conditioning exercise were inversely associated with T2D risk in age-adjusted and multivariable-adjusted analyses in both cohorts. However, when additionally adjusting for aerobic MVPA and mutually adjusting for resistance exercise and lower intensity muscular conditioning exercise, the association was attenuated for lower intensity muscular conditioning exercise in the NHSII, although it was significantly associated with T2D risk in pooled analyses (0.91 [95% CI 0.86–0.96] per 60 min/week of lower intensity muscular conditioning exercise). On the basis of the multivariable-adjusted restricted cubic spline regression models, we observed that the associations of individual types of activity with T2D risk were linear or had a slightly steeper gradient at less time spent on activity in each cohort of women (Figures 1–6), though the statistical tests for linearity provided no or weak evidence for a nonlinear association (*p*>0.05 for non-linear associations except for resistance exercise in NHSII [*p* = 0.02]). When we restricted the analyses to women reporting no aerobic activity, engagement in muscle-strengthening and conditioning activity was associated with lower risk of T2D in both cohorts of women (0.85 [95% CI 0.77–0.95] per 60 min/week in the pooled analysis) (Table 3). When we additionally adjusted for BMI, the association of muscle-strengthening and/or conditioning activities with T2D risk persisted (Table 2, model 3). Further adjustment for history of hypertension and raised cholesterol did not materially affect the results in either cohort of women (pooled RR's across categories of muscle-strengthening and conditioning activity were 0.86, 0.94, 0.79, and 0.63 [*p*<0.001 for trend]) (Table 4). Using a 2-year lag between exposure and T2D incidence for muscle-strengthening and conditioning activity did not materially change the results (Table S1). Repeating the analyses using the simple updated activity time instead of the cumulative average provided similar results (Table S2).

**Table 2:** Muscle-strengthening and conditioning activities and risk of type 2 diabetes in women from the Nurses' Health Study (2000–2008) and Nurses' Health Study II (2001–2009).

Characteristics	Minutes/Week of Activity						
	None	1–29	30–59	60–150	>150	*p*-trend	RR per 60 min/week
**Nurses' Health Study**							
**Total muscle-strengthening and conditioning activities**							
Median time (minutes/week)	0	12	39	95	225		
Cases/person-years	1,350/187,879	290/47,094	233/39,127	219/48,249	66/23,402		
Age adjusted	1.00	0.84 (0.74–0.95)	0.80 (0.70–0.92)	0.61 (0.53–0.70)	0.38 (0.29–0.48)	<0.001	0.81 (0.77–0.85)
Multivariable adjusted model 1^a^	1.00	0.86 (0.76–0.98)	0.87 (0.76–1.00)	0.70 (0.60–0.80)	0.46 (0.36–0.60)	<0.001	0.85 (0.81–0.89)
Multivariable adjusted model 2^b^	1.00	0.90 (0.79–1.02)	0.97 (0.83–1.11)	0.81 (0.70–0.94)	0.58 (0.45–0.75)	<0.001	0.90 (0.86–0.94)
Multivariable adjusted model 3^c^	1.00	0.94 (0.82–1.07)	1.03 (0.89–1.18)	0.89 (0.77–1.03)	0.63 (0.49–0.81)	<0.001	0.90 (0.85–0.96)
**Resistance exercise**							
Median time (minutes/week)	0	12	39	87	300		
Cases/person years	1,758/254,051	168/34,255	116/22,473	97/27,837	19/7,136		
Age adjusted	1.00	0.69 (0.58–0.80)	0.71 (0.58–0.85)	0.48 (0.39–0.59)	0.36 (0.23–0.57)	<0.001	0.76 (0.70–0.82)
Multivariable adjusted model 1^a^	1.00	0.74 (0.63–0.87)	0.80 (0.66–0.97)	0.58 (0.47–0.71)	0.47 (0.30–0.73)	<0.001	0.82 (0.76–0.89)
Multivariable adjusted model 2^b^	1.00	0.82 (0.70–0.97)	0.97 (0.80–1.18)	0.74 (0.59–0.91)	0.68 (0.43–1.07)	0.008	0.92 (0.86–0.99)
Multivariable adjusted model 3^c^	1.00	0.89 (0.75–1.05)	1.04 (0.85–1.26)	0.77 (0.62–0.95)	0.68 (0.43–1.08)	0.02	0.94 (0.87–1.01)
**Lower intensity muscular conditioning exercises**							
Median time (minutes/week)	0	12	39	81	300		
Cases/person years	1,524/219,870	258/45,395	236/42,925	121/31,269	19/6,291		
Age adjusted	1.00	0.80 (0.70–0.91)	0.77 (0.67–0.89)	0.55 (0.46–0.66)	0.43 (0.27–0.68)	<0.001	0.79 (0.73–0.84)
Multivariable adjusted model 1^a^	1.00	0.82 (0.72–0.94)	0.85 (0.74–0.98)	0.62 (0.52–0.75)	0.49 (0.31–0.77)	<0.001	0.83 (0.78–0.90)
Multivariable adjusted model 2^b^	1.00	0.88 (0.76–1.00)	1.00 (0.86–1.15)	0.76 (0.63–0.92)	0.62 (0.39–0.98)	0.004	0.91 (0.85–0.97)
Multivariable adjusted model 3^c^	1.00	0.89 (0.78–1.03)	1.05 (0.91–1.21)	0.82 (0.67–0.99)	0.65 (0.41–1.02)	0.02	0.93 (0.87–0.99)
**Nurses' Health Study II**							
**Total muscle-strengthening activities**							
Median time (minutes/week)	0	12	39	95	226		
Cases/person years	785/158,750	202/61,213	145/43,124	137/61,454	64/35,577		
Age adjusted	1.00	0.65 (0.55–0.75)	0.64 (0.54–0.77)	0.44 (0.36–0.52)	0.36 (0.27–0.46)	<0.001	0.79 (0.75–0.84)
Multivariable adjusted model 1^a^	1.00	0.69 (0.59–0.81)	0.72 (0.60–0.86)	0.51 (0.42–0.61)	0.43 (0.33–0.57)	<0.001	0.84 (0.79–0.89)
Multivariable adjusted model 2^b^	1.00	0.75 (0.64–0.88)	0.86 (0.72–1.04)	0.67 (0.55–0.81)	0.62 (0.48–0.82)	<0.001	0.92 (0.88–0.97)
Multivariable adjusted model 3^c^	1.00	0.85 (0.72–0.99)	0.95 (0.79–1.15)	0.79 (0.65–0.96)	0.75 (0.58–0.99)	0.02	0.96 (0.91–1.01)
**Resistance exercise**							
Median time (minutes/week)	0	12	39	98	300		
Cases/person years	998/213,288	129/53,072	78/30,571	105/49,300	23/13,887		
Age adjusted	1.00	0.51 (0.43–0.61)	0.50 (0.40–0.63)	0.46 (0.37–0.56)	0.36 (0.24–0.54)	<0.001	0.75 (0.69–0.81)
Multivariable adjusted model 1^a^	1.00	0.58 (0.48–0.69)	0.59 (0.46–0.74)	0.55 (0.45–0.67)	0.44 (0.29–0.67)	<0.001	0.81 (0.75–0.88)
Multivariable adjusted model 2^b^	1.00	0.68 (0.56–0.83)	0.77 (0.60–0.98)	0.77 (0.62–0.96)	0.70 (0.45–1.07)	0.03	0.94 (0.87–1.01)
Multivariable adjusted model 3^c^	1.00	0.76 (0.63–0.93)	0.85 (0.66–1.08)	0.87 (0.70–1.09)	0.78 (0.51–1.21)	0.21	0.96 (0.90–1.03)
**Lower intensity muscular conditioning exercises**							
Median time (minutes/week)	0	12	39	75	300		
Cases/person years	908/206,956	199/64,454	141/53,843	74/29,640	11/5,225		
Age adjusted	1.00	0.67 (0.57–0.78)	0.58 (0.49–0.69)	0.53 (0.42–0.68)	0.43 (0.24–0.78)	<0.001	0.70 (0.63–0.78)
Multivariable adjusted model 1^a^	1.00	0.72 (0.61–0.84)	0.65 (0.55–0.78)	0.61 (0.48–0.77)	0.48 (0.26–0.86)	<0.001	0.76 (0.69–0.85)
Multivariable adjusted model 2^b^	1.00	0.86 (0.73–1.01)	0.89 (0.74–1.08)	0.89 (0.69–1.14)	0.72 (0.39–1.31)	0.15	0.91 (0.83–1.01)
Multivariable adjusted model 3^c^	1.00	0.91 (0.77–1.07)	0.97 (0.80–1.17)	0.99 (0.77–1.27)	0.79 (0.43–1.45)	0.49	0.95 (0.87–1.04)
**Pooled results** d							
Multivariable adjusted model 2							
Total muscle-strengthening and conditioning activities	1.00	0.83 (0.75–0.92)	0.93 (0.83–1.04)	0.75 (0.67–0.85)	0.60 (0.50–0.72)	<0.001	0.91 (0.88–0.94)
Resistance exercise	1.00	0.76 (0.67–0.86)	0.89 (0.76–1.03)	0.75 (0.64–0.87)	0.69 (0.50–0.94)	<0.001	0.93 (0.88–0.98)
Lower intensity muscular conditioning exercises	1.00	0.87 (0.78–0.96)	0.96 (0.85–1.07)	0.80 (0.69–0.94)	0.65 (0.45–0.94)	0.002	0.91 (0.86–0.96)
Multivariable adjusted model 3							
Total muscle-strengthening and conditioning activities	1.00	0.90 (0.81–1.00)	1.00 (0.89–1.12)	0.86 (0.77–0.96)	0.86 (0.75–0.98)	<0.001	0.94 (0.91–0.97)
Resistance exercise	1.00	0.83 (0.73–0.94)	0.96 (0.82–1.11)	0.82 (0.70–0.95)	0.74 (0.54–1.01)	0.01	0.95 (0.90–1.00)
Lower intensity muscular conditioning exercises	1.00	0.90 (0.81–1.00)	1.02 (0.91–1.14)	0.88 (0.75–1.02)	0.70 (0.48–1.00)	0.03	0.94 (0.89–0.99)

Data are relative risks (95% CI).

^a^ Adjusted for age (months), smoking (never, past, or current), alcohol consumption (0, 1–5, >5 g/d), coffee intake (0, <1, 1–3, 3–5, >5 cups/day), race (white, non-white), family history of diabetes, post menopausal hormone use (never, past, current), intake of total energy, trans fat, polyunsaturated fat to saturated fat ratio, cereal fiber, wholegrain, and glycemic load (all dietary factors in quintiles), oral contraceptive use (only NHSII: never, past, current), menopausal status (only NHSII: pre, post).

^b^ Additionally adjusted for aerobic physical activity (categorized similar to muscle-strengthening activities), and resistance exercise and lower intensity muscular conditioning exercises were mutually adjusted.

^c^ Additionally adjusted for BMI (continuous).

^d^ Combined using fixed effect pooling.

**Table 3:** Total muscle-strengthening and conditioning activities and risk of type 2 diabetes restricted to women who did not engage in aerobic activity.

Study	*n*	Person-Years	Cases	RR per 60 Min/Week
**Nurses' Health Study I**	17,429	93,616	859	0.85 (0.77–0.95)
**Nurses' Health Study II**	11,535	67,877	466	0.78 (0.64–0.95)
**Pooled results** a	—	—	—	0.84 (0.76–0.92)

Data are relative risks (95% CI). Multivariable model included age (months), smoking (never, past, or current), alcohol consumption (0, 1–5, >5 g/d), coffee intake (0, <1, 1–3, 3–5, >5 cups/day), race (white, non-white), family history of diabetes, post menopausal hormone use (never, past, current), intake of total energy, trans fat, polyunsaturated fat to saturated fat ratio, cereal fiber, wholegrain, and glycemic load (all dietary factors in quintiles), oral contraceptive use (only NHSII: never, past, current), menopausal status (only NHSII: pre, post).

^a^ Combined using fixed effect pooling of estimates from multivariable adjusted models.

**Table 4:** Total muscle-strengthening and conditioning activities and risk of type 2 diabetes in women from the Nurses' Health Study and Nurses' Health Study II with additional adjustment for history of hypertension and raised cholesterol.

Study	Minutes/Week of Muscle-Strengthening Activity					
	None	1–29	30–59	60–150	>150	*p*-trend
**Nurses' Health Study**						
Cases/person years	1,350/187,879	290/47,094	233/39,127	219/48,249	66/23,402	
Multivariable adjusted model^a^	1.00	0.91 (0.80–1.04)	0.98 (0.85–1.13)	0.84 (0.72–0.97)	0.60 (0.46–0.77)	<0.001
**Nurses' Health Study II**						
Cases/person years	785/158,750	202/61,213	145/43,124	137/61,454	64/35,577	
Multivariable adjusted model^a^	1.00	0.79 (0.67–0.93)	0.91 (0.76–1.09)	0.71 (0.58–0.86)	0.63 (0.48–0.84)	<0.001
**Pooled results** b	1.00	0.86 (0.78–0.95)	0.94 (0.84–1.06)	0.79 (0.70–0.88)	0.63 (0.52–0.75)	<0.001

Data are relative risks (95% CI).

^a^ Adjusted for age (months), smoking (never, past, or current), alcohol consumption (0, 1–5, >5 g/d), coffee intake (0, <1, 1–3, 3–5, >5 cups/day), race (white, non-white), family history of diabetes, post menopausal hormone use (never, past, current), intake of total energy, trans fat, polyunsaturated fat to saturated fat ratio, cereal fiber, wholegrain, and glycemic load (all dietary factors in quintiles), oral contraceptive use (only NHSII: never, past, current), menopausal status (only NHSII: pre, post), aerobic physical activity (categorized similar to muscle-strengthening activities), history of hypertension and raised cholesterol.

^b^ Combined using fixed effect pooling.

The stratified analyses by BMI indicated that engagement in muscle-strengthening activity was associated with lower T2D risk among overweight (BMI 25 to <30 kg/m^2^) and obese (≥30 kg/m^2^) women, but no association was observed among normal weight women (BMI<25 kg/m^2^) (Table 5). There was no evidence that the association of muscle-strengthening and conditioning activity with T2D risk was different across age (<65, ≥65 years, NHS only), family history of T2D, diet quality score, race (white, non-white), and aerobic MVPA (quintiles) in either cohort of women (*p*>0.05 for multiplicative interaction) (Table S3).

**Table 5:** Total muscle-strengthening and conditioning activities and risk of type 2 diabetes stratified by body mass index (<25, 25–<30, ≥30 kg/m^2^).

BMI Category	Cases/PY	Minutes per Week of Muscle-Strengthening and Conditioning Activity			*p* for Trend	RR per 60 min/day
		None	>0–59 min	≥60 min		
**Nurses' Health Study**						
<25.0 kg/m^2^	316/162,304	1.00	0.90 (0.68–1.18)	1.00 (0.74–1.35)	0.87	0.97 (0.89–1.07)
25.0–29.9 kg/m^2^	725/118,072	1.00	0.92 (0.77–1.10)	0.71 (0.57–0.90)	0.005	0.89 (0.82–0.97)
≥30.0 kg/m^2^	1,118/65,456	1.00	1.08 (0.93–1.25)	0.86 (0.70–1.04)	0.11	0.94 (0.88–1.01)
**Nurses' Health Study II**						
<25.0 kg/m^2^	78/174,933	1.00	1.04 (0.59–1.82)	0.99 (0.54–1.87)	0.97	1.00 (0.87–1.14)
25.0–29.9 kg/m^2^	212/101,634	1.00	1.19 (0.86–1.63)	0.79 (0.53–1.19)	0.14	0.96 (0.87–1.06)
≥30.0 kg/m^2^	1,043/83,551	1.00	0.84 (0.72–0.98)	0.85 (0.70–1.04)	0.16	0.98 (0.92–1.04)
**Pooled**						
<25.0 kg/m^2^	—	1.00	0.92 (0.72–1.18)	1.00 (0.76–1.31)	0.90	0.98 (0.91–1.06)
25.0–29.9 kg/m^2^	—	1.00	0.98 (0.84–1.15)	0.73 (0.60–0.90)	0.002	0.92 (0.86–0.98)
≥30.0 kg/m^2^	—	1.00	0.96 (0.86–1.06)	0.85 (0.74–0.98)	0.03	0.96 (0.92–1.01)

Data are relative risks (95% CI). Adjusted for age (months), smoking (never, past, or current), alcohol consumption (0, 1–5, >5 g/d), coffee intake (0, <1, 1–3, 3–5, >5 cups/day), race (white, non-white), family history of diabetes, post menopausal hormone use (never, past, current), intake of total energy, trans fat, polyunsaturated fat to saturated fat ratio, cereal fiber, wholegrain, and glycemic load (all dietary factors in quintiles), oral contraceptive use (only NHSII: never, past, current), menopausal status (only NHSII: pre, post), and aerobic physical activity (none, 1–29, 30–59, 60–150, >150 min/week).

PY, person years.

Aerobic MVPA was inversely associated with T2D risk in the multivariable model after adjustment for resistance exercise, lower intensity muscular conditioning exercise, and BMI in both cohorts (*p*<0.001 for trend) (Table 6). Spline regression revealed that the association of aerobic MVPA with the risk T2D was non-linear in both cohorts, with the steepest gradient at lower levels of activity (*p*<0.01) (Figures S1 and S2).

**Table 6:** Aerobic moderate-and-vigorous physical activity and risk of type 2 diabetes in women from the Nurses' Health Study (2000–2008) and Nurses' Health Study II (2001–2009).

Characteristics	Minutes/week of aerobic moderate-and-vigorous physical activity					
	None	1–29	30–59	60–150	>150	*p* Trend
**Nurses' Health Study**						
Median time (minutes/week)	0	13	41	79	300	
Cases/person years	859/93,616	245/32,151	238/35,047	379/64,802	437/120,136	
Multivariable adjusted model 1	1.00	0.84 (0.73–0.97)	0.76 (0.66–0.88)	0.68 (0.60–0.77)	0.48 (0.42–0.54)	<0.001
Multivariable adjusted model 2	1.00	0.94 (0.81–1.09)	0.88 (0.76–1.02)	0.85 (0.74–0.96)	0.66 (0.58–0.75)	<0.001
**Nurses' Health Study II**						
Median time (minutes/week)						
Cases/person years	466/67,881	182/36,430	162/39,723	259/77,606	264/138,478	
Multivariable adjusted model 1	1.00	0.80 (0.67–0.95)	0.68 (0.57–0.82)	0.63 (0.54–0.74)	0.42 (0.36–0.50)	<0.001
Multivariable adjusted model 2	1.00	0.94 (0.79–1.13)	0.83 (0.69–1.00)	0.86 (0.73–1.01)	0.70 (0.59–0.83)	<0.001
**Pooled results** *						
Multivariable adjusted model 1	1.00	0.83 (0.74–0.92)	0.73 (0.65–0.82)	0.66 (0.60–0.73)	0.46 (0.41–0.50)	<0.001
Multivariable adjusted model 2	1.00	0.94 (0.84–1.05)	0.86 (0.77–0.97)	0.85 (0.77–0.94)	0.67 (0.61–0.75)	<0.001

Data are relative risks (95% CI). Model 1 was adjusted for age (months), smoking (never, past, or current), alcohol consumption (0, 1–5, >5 g/d), coffee intake (0, <1, 1–3, 3–5, >5 cups/day), race (white, non-white), family history of diabetes, post menopausal hormone use (never, past, current), intake of total energy, trans fat, polyunsaturated fat to saturated fat ratio, cereal fiber, wholegrain, and glycemic load (all dietary factors in quintiles), oral contraceptive use (only NHSII: never, past, current), menopausal status (only NHSII: pre, post), resistance exercise (0, 1–29, 30–59, 60–150, >150 min/week) and lower intensity muscular conditioning exercises (0, 1–29, 30–59, 60–150, >150 min/week). Model 2 was additionally adjusted for BMI (continues). Aerobic MVPA consists of brisk walking, jogging, running, bicycling, tennis, swimming, other aerobic exercise, other vigorous exercise, and stair climbing.

Combined using fixed effect pooling of estimates from multivariable adjusted models.

**Figure 7:**
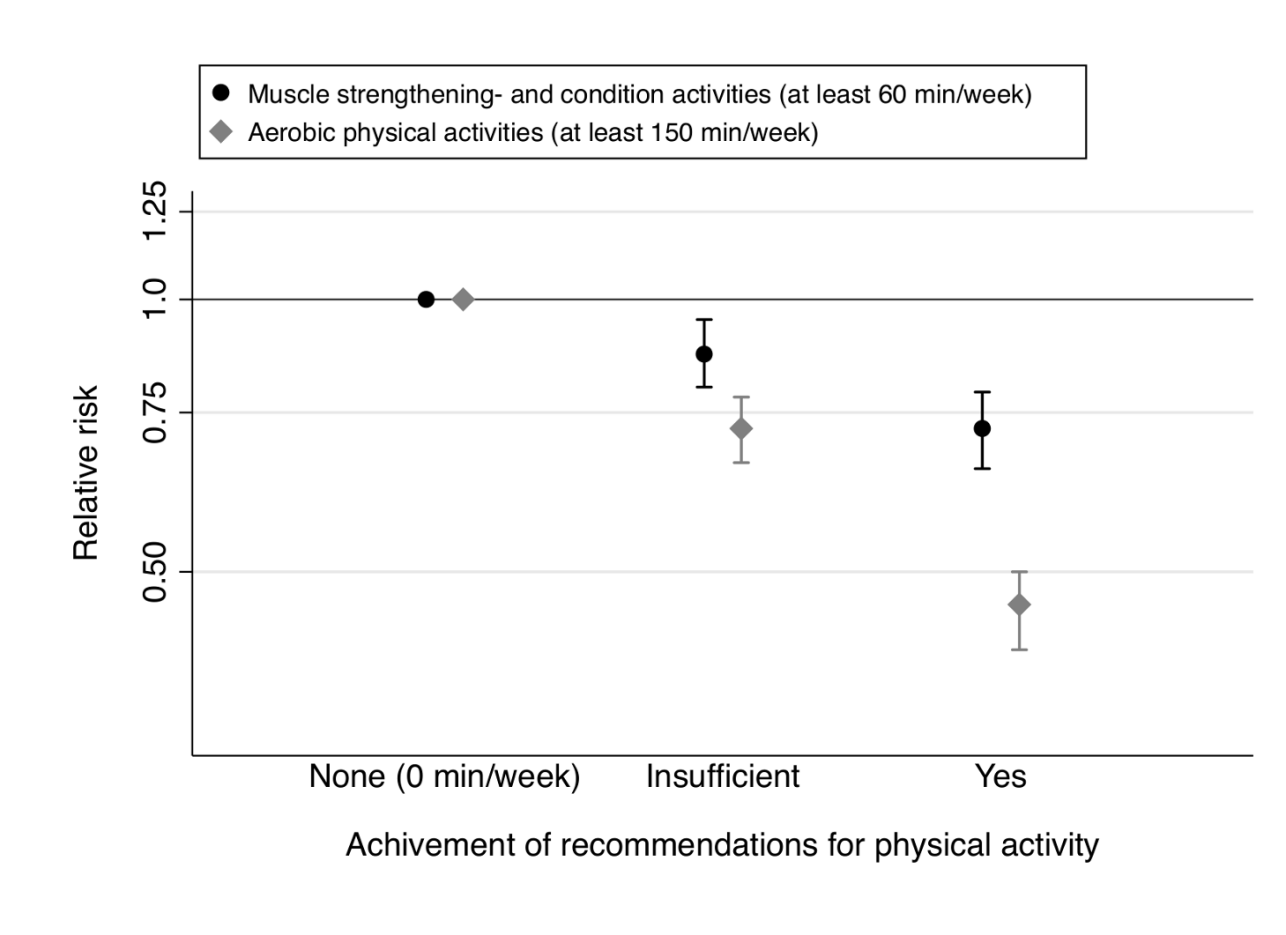
Muscle-strengthening and aerobic activity according to recommendations [13]–[15] and type 2 diabetes risk. Women in the category “Insufficient” engage in some activity but less than recommended. Data are pooled estimates (fixed effect) of RR's with 95% CI from multivariable cox regression models adjusted for age (months), smoking (never, past, or current), alcohol consumption (0, 1–5, >5 g/d), coffee intake (0, <1, 1–3, 3–5, >5 cups/day), race (white, non-white), family history of diabetes, post menopausal hormone use (never, past, current), intake of total energy, trans fat, polyunsaturated fat to saturated fat ratio, cereal fiber, wholegrain, and glycemic load (all dietary factors in quintiles), oral contraceptive use (only NHSII: never, past, current), menopausal status (only NHSII: pre, post). Muscle-strengthening and conditioning activities and aerobic MVPA were mutually adjusted.

Achievement of recommendations [13]–[15] for muscle-strengthening and conditioning activities (none/no/yes) and aerobic MVPA (none/no/yes) was each independently associated with lower T2D risk in multivariable adjusted analysis: the pooled RR was 0.46 (95% CI 0.41–0.50) for aerobic MVPA and 0.72 (95% CI 0.65–0.79) for muscle-strengthening and conditioning activities compared with women reporting no activity (Figure 7). Furthermore, compared with women reporting no activity, engagement in a level of activity that is less than the recommended, of either muscle-strengthening type or aerobic MVPA, was associated with a lower T2D risk (pooled RR = 0.72 (95% CI 0.66–0.78) for aerobic MVPA and pooled RR = 0.87 (95% CI 0.80–0.95) for muscle-strengthening and conditioning activities). In the joint association analysis, women who adhered to the recommendations for both aerobic MVPA and resistance exercise had the lowest risk of T2D risk in the multivariable model, with a pooled RR of 0.33 (95% CI 0.29–0.38) compared with women who were inactive (Table 7).

**Table 7:** Joint association of total muscle-strengthening and conditioning activities and aerobic MVPA according to recommendations [13]–[15] and risk of type 2 diabetes in women from the Nurses' Health Study (2000–2008) and Nurses' Health Study II (2001–2009).

Characteristics	None(No Aerobic MVPA and No Muscle-Strengthening Activity)	Neither Recommendation(Engage in Some Activity but Less Than Recommended)	Recommendation for Muscle-Strengthening Activities only(≥60 min/week)	Recommendation for Aerobic MVPA only(≥150 min/week)	Recommendation for Both Muscle-Strengthening Activities and Aerobic MVPA
**Nurses' Health Study**					
Cases/person years	672/69,052	851/120,358	193/35,507	305/76,975	137/43,861
Age adjusted	1.00	0.70 (0.63–0.78)	0.54 (0.46–0.63)	0.39 (0.34–0.45)	0.30 (0.25–0.36)
Multivariable adjusted model	1.00	0.73 (0.66–0.81)	0.60 (0.51–0.70)	0.45 (0.39–0.51)	0.37 (0.31–0.45)
**Nurses' Health Study II**					
Cases/person years	383/52,285	570/132,689	116/36,578	156/70,641	108/67,924
Age adjusted	1.00	0.57 (0.50–0.65)	0.41 (0.33–0.50)	0.30 (0.25–0.37)	0.22 (0.18–0.27)
Multivariable adjusted model	1.00	0.63 (0.55–0.72)	0.46 (0.38–0.57)	0.37 (0.31–0.45)	0.29 (0.23–0.36)
**Pooled results** a	1.00	0.69 (0.64–0.75)	0.54 (0.48–0.62)	0.42 (0.38–0.47)	0.33 (0.29–0.38)

Muscle-strengthening and conditioning activities were defined as engaging in at least two times/week (we approximated this to be equivalent to ≥60 min/week). Aerobic MVP was defined as ≥150 min/week of at least moderate intensity. Data are relative risks (95% CI). Multivariable model included age (months), smoking (never, past, or current), alcohol consumption (0, 1–5, >5 g/d), coffee intake (0, <1, 1–3, 3–5, >5 cups/day), race (white, non-white), family history of diabetes, post menopausal hormone use (never, past, current), intake of total energy, trans fat, polyunsaturated fat to saturated fat ratio, cereal fiber, wholegrain, and glycemic load (all dietary factors in quintiles), oral contraceptive use (only NHSII: never, past, current), menopausal status (only NHSII: pre, post).

^a^ Combined using fixed effect pooling of estimates from multivariable adjusted models.

## Discussion

In this prospective study of nearly 100,000 women from the NHS I and II, regular participation in muscle-strengthening and conditioning activities, such as resistance exercise and lower intensity muscular conditioning exercise, was associated with a reduced risk of T2D, fairly consistent with a dose-response relationship independent of aerobic MVPA and other major determinants for T2D including BMI. The findings from our study also suggest that incorporating muscle-strengthening and conditioning activities with aerobic activity according to the current recommendation for physical activity [13]–[15] provides substantial benefit for T2D prevention in women.

We are not aware of any prior work reporting associations of muscle-strengthening or conditioning activities with T2D risk in women. Our findings extends those from our previous study among men from the Health Professional Follow-up Study (HPFS) [10]. Our results are also supported by a recent cross-sectional study among Australian adults reporting that regular strength training was associated with lower prevalence of impaired glucose metabolism independent of MVPA [24]. Furthermore, our results are consistent with a meta-analysis of findings from randomized controlled trials showing that resistance training can improve glycemic control in the absence of aerobic activity among individuals with T2D [9]. In addition, a recent study showed that a single session of aerobic or resistance exercise yielded similar effects on 24 hours post-exercise glycemic control in insulin-resistant individuals with and without T2D [25]. Thus, the cumulative evidence from observational and experimental studies supports that muscle-strengthening activities can serve as an alternative to aerobic exercise for T2D prevention and glycemic control.

Engagement in lower intensity muscle conditioning exercise such as yoga for T2D prevention has to our knowledge not previously been studied. However, a number of randomized clinical trials of mixed quality on participation in yoga intervention for management of T2D have been carried out. The majority of these studies suggest that yoga can improve glycemic control among individuals with T2D [26]–[29]. The three largest trials were completed recently and were of moderate size (*n* = 59–123) [26],[28],[29]. Two of these studies found modest improvement in glycemic control after a yoga intervention for three or six months compared with standard care [28],[29]. A study among 59 individuals with T2D found no significant benefits of yoga intervention for 12 weeks on glycemic control compared with a waiting list control group [26]. However, an adherence rate of only 50% was reported in the yoga intervention group. We obtained information on lower-intensity muscle conditioning (yoga, stretching, toning) from a single question and we were not able to separate, e.g., yoga exercise from toning and stretching. Yoga practice and toning exercise are likely to vary in intensity and type between individuals, and more detailed information on these types of activities would have been useful. A previous study has reported energy expenditure during yoga sessions to being of low intensity equivalent to slow walking [30].

Two large randomized trials among individuals with T2D also suggest that the combination of aerobic exercise and resistance training results in greatest improvement in glycemic control compared with either type of activity alone [3],[4]. Our results from the current study and our previous report in men also support the recommendation to include muscle-strengthening and conditioning activities beyond participation in aerobic MVPA [10]. We did not see indications that the association of muscle-strengthening and conditioning activities with T2D risk was modified by time spent on aerobic MVPA, which suggest that it may be beneficial to add muscle-strengthening and conditioning activity even at higher levels of aerobic MVPA for T2D prevention.

Resistance exercise may decrease the risk of T2D through several mechanisms. Because aging is associated with increasing loss of lean body mass, one important role may be the effect of muscle-strengthening and conditioning activity on skeletal muscle mass maintenance [31]. However, there are possibly also effects of these exercise types extrinsic to muscle mass growth. Other effects reported for resistance exercise include increases in glycolytic capacity and up-regulation of proteins in the insulin-signaling cascade [32]. These local adaptations are likely to result in increases in insulin action and enhancement of the capacity for glucose utilization. Randomized trials have also shown that resistance exercise alone can improve blood pressure, lipid levels, and reduce adiposity [33]. We found that adiposity indicated by BMI only partly explained the association of muscle-strengthening and conditioning activities with T2D risk, suggesting that participation in these types of activities can lower T2D risk without changing body weight. However, BMI is unable to distinguish fat mass from fat-free mass, and engagement in muscle-strengthening activity is likely to increase fat-free mass while decreasing fat mass. We also observed greater risk reduction with participation in muscle-strengthening and conditioning activity among overweight and obese women and no apparent effect among normal weight women. This may suggest that these types of activities have less effect in terms of T2D prevention among women who already maintain a healthy weight.

A limitation of the present study includes that the study population consisted of registered nurses with mostly European ancestry. It is therefore unknown if our results can be generalized to other populations of women. Physical activity was assessed by a self-administered questionnaire and is therefore prone to misclassification. While our validation study among a random sample of *n* = 147 NHSII participants described a moderate to strong (r = 0.62) relationship between total physical activity as reported in the questionnaire and that reported in four one-week diaries, we did not obtain specific validation data on muscle-strengthening activities, and the validity of the self-reported time spent on these activities remains uncertain in our cohorts. We do not expect differential misclassification of these activities by subsequent incident T2D and the estimated associations of activity with T2D are therefore likely to be underestimated. Because we updated physical activity during follow-up, the expected genuine individual variation in physical activity over time is better accounted for, which would avoid further dilution bias of estimated associations. Furthermore, residual and unknown confounding cannot be fully excluded, as the present study is observational. As we observed risk reduction with muscle-strengthening and conditioning activity among women reporting no aerobic activity, this reassures us that the associations we observed are not likely to be explained by residual confounding by aerobic MVPA. The strengths of the study include the large sample size, updated information on activity and other covariates, and that we were able to control for a wide range of confounding factors. Furthermore, the results were robust to excluding T2D cases during the first two years of follow-up and using only the baseline information on muscle-strengthening activity.

In summary, this large prospective study of US women suggests that engagement in muscle-strengthening and conditioning activities can lower the risk of T2D independent of aerobic MVPA. Following the recommendations for both muscle-strengthening activities and aerobic MVPA was associated with a substantial reduction in the risk of T2D. Engagement in levels lower than currently recommended of aerobic physical activity and muscle-strengthening and conditioning activity were also significantly associated with lower T2D risk. Collectively, our study supports the inclusion of muscle-strengthening and conditioning activities in preventive measures against T2D, consistent with the current guidelines for physical activity among adults.

## Supporting Information

Figure S1 **Dose-response relationship between aerobic physical activity (hours/week) and risk of type 2 diabetes in women from the Nurses' Health Study.** Dotted lines are 95% CI for the trend obtained from restriced cubic spline regression (4 knots) truncated at 12.5 hours/week (≈99th percentile). Estimates were adjusted for age (months), smoking (never, past, or current), alcohol consumption (0, 1–5, >5 g/d), coffee intake (0, <1, 1–3, 3–5, >5 cups/day), race (white, non-white), family history of diabetes, post menopausal hormone use (never, past, current), intake of total energy, trans fat, polyunsaturated fat to saturated fat ratio, cereal fiber, wholegrain, and glycemic load (all dietary factors in quintiles), resistance exercise (0, 1–29, 30–59, 60–150, >150 min/week) and lower intensity muscular conditioning exercises (0, 1–29, 30–59, 60–150, >150 min/week). *p*<0.001 for non-linear response.
(TIF)

Figure S2 **Dose-response relationship between aerobic physical activity (hours/week) and risk of type 2 diabetes in women from the Nurses' Health Study II.** Dotted lines are 95% CI for the trend obtained from restriced cubic spline regression (4 knots) truncated at 12.5 hours/week (≈99th percentile). Estimates were adjusted for age (months), smoking (never, past, or current), alcohol consumption (0, 1–5, >5 g/d), coffee intake (0, <1, 1–3, 3–5, >5 cups/day), race (white, non-white), family history of diabetes, post menopausal hormone use (never, past, current), intake of total energy, trans fat, polyunsaturated fat to saturated fat ratio, cereal fiber, wholegrain, and glycemic load (all dietary factors in quintiles), oral contraceptive use (never, past, current), menopausal status (pre, post), resistance exercise (0, 1–29, 30–59, 60–150, >150 min/week) and lower intensity muscular conditioning exercises (0, 1–29, 30–59, 60–150, >150 min/week). *p*<0.001 for non-linear response.
(TIF)

Table S1 **Total muscle-strengthening activities and risk of type 2 diabetes in women from the Nurses' Health Study and Nurses' Health Study II using a 2-year lag between exposure and incidence of T2D.** Data are relative risks (95% CI). *adjusted for age (months), smoking (never, past, or current), alcohol consumption (0, 1–5, >5 g/d), coffee intake (0, <1, 1–3, 3–5, >5 cups/day), race (white, non-white), family history of diabetes, post menopausal hormone use (never, past, current), intake of total energy, trans fat, polyunsaturated fat to saturated fat ratio, cereal fiber, wholegrain, and glycemic load (all dietary factors in quintiles), oral contraceptive use (only NHSII: never, past, current), menopausal status (only NHSII: pre, post), aerobic physical activity (categorized similar to muscle-strengthening activities). ** Combined using fixed effect pooling.
(DOCX)

Table S2 **Muscle-strengthening and conditioning activities and risk of type 2 diabetes in women from the Nurses' Health Study (2000–2008) and Nurses' Health Study II (2001–2009) based on the simple updated activity level (recent activity level).** All analyses were adjusted for age (months), smoking (never, past, or current), alcohol consumption (0, 1–5, >5 g/d), coffee intake (0, <1, 1–3, 3–5, >5 cups/day), race (white, non-white), family history of diabetes, post menopausal hormone use (never, past, current), intake of total energy, trans fat, polyunsaturated fat to saturated fat ratio, cereal fiber, wholegrain, and glycemic load (all dietary factors in quintiles), oral contraceptive use (only NHSII: never, past, current), menopausal status (only NHSII: pre, post), aerobic physical activity (categorized similar to muscle-strengthening activities). Estimates of associations for resistance exercise and lower intensity muscular conditioning exercises were mutually adjusted for each other. *Combined using fixed effect pooling.
(DOCX)

Table S3 **Muscle-strengthening activities and risk of type 2 diabetes stratified by age (<65, ≥65 years, NHS only), family history of T2D, diet quality, race (white, non-white), and aerobic physical activity (quintiles).** Data are relative risks (95% CI). Multivariable model included age (months), smoking (never, past, or current), alcohol consumption (0, 1–5, >5 g/d), coffee intake (0, <1, 1–3, 3–5, >5 cups/day), race (white, non-white), family history of diabetes, post menopausal hormone use (never, past, current), intake of total energy, trans fat, polyunsaturated fat to saturated fat ratio, cereal fiber, wholegrain, and glycemic load (all dietary factors in quintiles), oral contraceptive use (only NHSII: never, past, current), menopausal status (only NHSII: pre, post), aerobic physical activity (none, 1–29, 30–59, 60–150, >150 min/week). *Only adjusted for age and aerobic physical activity (none, 1–29, 30–59, 60–150, >150 min/week) due to the low number of cases in some groups.
(DOCX)

## Abbreviations

BMI: body mass index
MVPA: moderate and vigorous physical activity
Nurses' Health Study: NHS
Nurses' Health Study II: NHSII
T2D: type 2 diabetes

## References

[1] Hu FB, Leitzmann MF, Stampfer MJ, Colditz GA, Willett WC, et al (2001) Physical activity and television watching in relation to risk for type 2 diabetes mellitus in men. Arch Intern Med 161: 1542–1548. 11427103 10.1001/archinte.161.12.1542

[2] Hu FB, Li TY, Colditz GA, Willett WC, Manson JE (2003) Television watching and other sedentary behaviors in relation to risk of obesity and type 2 diabetes mellitus in women. JAMA 289: 1785–1791. 12684356 10.1001/jama.289.14.1785

[3] Sigal RJ, Kenny GP, BoulÇ NG, Wells GA, Prud'homme D, et al (2007) Effects of aerobic training, resistance training, or both on glycemic control in type 2 diabetes. Ann Intern Med 147: 357–369. 17876019 10.7326/0003-4819-147-6-200709180-00005

[4] Church TS, Blair SN, Cocreham S, Johannsen N, Johnson W, et al (2010) Effects of aerobic and resistance training on hemoglobin a1c levels in patients with type 2 diabetes. JAMA 304: 2253–2262. 21098771 10.1001/jama.2010.1710 PMC3174102

[5] Dunstan DW, Daly RM, Owen N, Jolley D, de C, et al (2002) High-intensity resistance training improves glycemic control in older patients with type 2 diabetes. Diabetes Care 25: 1729–1736. 12351469 10.2337/diacare.25.10.1729

[6] Bacchi E, Negri C, Zanolin ME, Milanese C, Faccioli N, et al (2012) Metabolic effects of aerobic training and resistance training in type 2 diabetic subjects. Diabetes Care 35: 676–682. 22344613 10.2337/dc11-1655 PMC3308269

[7] Balducci SZSNA, et al (2010) Effect of an intensive exercise intervention strategy on modifiable cardiovascular risk factors in subjects with type 2 diabetes mellitus: a randomized controlled trial: the Italian diabetes and exercise study (ides). Arch Intern Med 170: 1794–1803. 21059972 10.1001/archinternmed.2010.380

[8] Castaneda C, Layne JE, Munoz-Orians L, Gordon PL, Walsmith J, et al (2002) A randomized controlled trial of resistance exercise training to improve glycemic control in older adults with type 2 diabetes. Diabetes Care 25: 2335–2341. 12453982 10.2337/diacare.25.12.2335

[9] Umpierre D, Ribeiro PAB, Kramer CK, Leitao CB, Zucatti ATN, et al (2011) Physical activity advice only or structured exercise training and association with HbA1c levels in type 2 diabetes. JAMA 305: 1790–1799. 21540423 10.1001/jama.2011.576

[10] Grøntved A, Rimm EB, Willett WC, Andersen LB, Hu FB (2012) A prospective study of weight training and risk of type 2 diabetes in men. Arch Intern Med 172: 1306–1312. 22868691 10.1001/archinternmed.2012.3138 PMC3822244

[11] Roland KP, Jakobi JM, Jones GR (2011) Does yoga engender fitness in older adults? A critical review. J Aging Phys Activ 19: 62–79. 10.1123/japa.19.1.62 21285476

[12] Cruz-Ferreira A, Fernandes J, Laranjo L, Bernardo LM, Silva A (2011) A systematic review of the effects of pilates method of exercise in healthy people. Arch Phys Med Rehab 92: 2071–2081. 10.1016/j.apmr.2011.06.018 22030232

[13] Colberg SR, Sigal RJ, Fernhall B, Regensteiner JG, Blissmer BJ, et al (2010) Exercise and type 2 diabetes. Diabetes Care 33: e147–e167. 21115758 10.2337/dc10-9990 PMC2992225

[14] (2008) Physical Activity Guidelines Advisory Committee Report. Washington, DC: USDepartment of Health and Human Services and 2008 Physical activity guidelines for Americans (http://www.health.gov/paguidelines/guidelines/default.aspx (accessed Oct 21st, 2013)).

[15] World Health Organization (2010) Global recommendations on physical activity for health. Geneva: WHO Press. 26180873

[16] Wolf AM, Hunter DJ, Colditz GA, Manson JE, Stampfer MJ, et al (1994) Reproducibility and validity of a self-administered physical activity questionnaire. Int J Epidemiol 23: 991–999. 7860180 10.1093/ije/23.5.991

[17] Chasan-Taber S, Rimm EB, Stampfer MJ, Spiegelman D, Colditz GA, et al (1995) Reproducibility and validity of a self-administered physical activity questionnaire for male health professionals. Epidemiology 7: 81–86. 10.1097/00001648-199601000-00014 8664406

[18] Report of the Expert Committee on the Diagnosis and Classification of Diabetes Mellitus. Diabetes Care 20: 1183–1197. 10.2337/diacare.20.7.1183 9203460

[19] Manson JE, Stampfer MJ, Colditz GA, Willett WC, Rosner B, et al (1991) Physical activity and incidence of non-insulin-dependent diabetes mellitus in women. Lancet 338: 774–778. 1681160 10.1016/0140-6736(91)90664-b

[20] Field AE, Coakley EH, Must A, Spadano JL, Laird N, et al (2001) Impact of overweight on the risk of developing common chronic diseases during a 10-year period. Arch Intern Med 161: 1581–1586. 11434789 10.1001/archinte.161.13.1581

[21] Willett WC, Sampson L, Stampfer MJ, Rosner B, Bain C, et al (1985) Reproducibility and validity of a semiquantitative food frequency questionnaire. Am J Epidemiol 122: 51–65. 4014201 10.1093/oxfordjournals.aje.a114086

[22] Hu FB, Manson JE, Stampfer MJ, Colditz G, Liu S, et al (2001) Diet, lifestyle, and the risk of type 2 diabetes mellitus in women. New Engl J Med 345: 790–797. 11556298 10.1056/NEJMoa010492

[23] Greenland S (1995) Dose-response and trend analysis in epidemiology: alternatives to categorical analysis. Epidemiology 6: 356–365. 7548341 10.1097/00001648-199507000-00005

[24] Minges KE, Magliano DJ, Owen N, Daly RM, Salmon J, et al (2012) Associations of strength training with impaired glucose metabolism: the AusDiab Study. Med Sci Sports Exerc 45: 299–303. 10.1249/MSS.0b013e31826e6cd1 22903138

[25] van Dijk JW, Manders R, Tummers K, Bonomi A, Stehouwer C, et al (2011) Both resistance- and endurance-type exercise reduce the prevalence of hyperglycaemia in individuals with impaired glucose tolerance and in insulin-treated and non-insulin-treated type 2 diabetic patients. Diabetologia 1–10. 22124605 10.1007/s00125-011-2380-5 PMC3331783

[26] Skoro-Kondza L, Tai S, Gadelrab R, Drincevic D, Greenhalgh T (2009) Community based yoga classes for type 2 diabetes: an exploratory randomised controlled trial. BMC Health Serv Res 9: 33. 19228402 10.1186/1472-6963-9-33 PMC2652459

[27] Monro R, Power J, Coumar A, Nagarathna R, Dandona P (1992) Yoga therapy for NIDDM: A controlled trial. Complementary Medical Research 6: 66–68.

[28] Gordon L, Morrison E, McGrowder D, Young R, Fraser Y, et al (2008) Effect of exercise therapy on lipid profile and oxidative stress indicators in patients with type 2 diabetes. BMC Complem Altern M 8: 21. 10.1186/1472-6882-8-21 PMC2390515 18477407

[29] Hegde SV, Adhikari P, Kotian S, Pinto VJ, D'Souza S, et al (2011) Effect of 3-month yoga on oxidative stress in type 2 diabetes with or without complications. Diabetes Care 34: 2208–2210. 21836105 10.2337/dc10-2430 PMC3177728

[30] Hagins M, Moore W, Rundle A (2007) Does practicing hatha yoga satisfy recommendations for intensity of physical activity which improves and maintains health and cardiovascular fitness? BMC Complem Altern M 7: 40. 10.1186/1472-6882-7-40 PMC2219995 18053143

[31] LeBrasseur NK, Walsh K, Arany Z (2011) Metabolic benefits of resistance training and fast glycolytic skeletal muscle. Am J Physiol Endocrinol Metab 300: E3–E10. 21045171 10.1152/ajpendo.00512.2010 PMC3023213

[32] Holten MK, Zacho M, Gaster M, Juel C, Wojtaszewski JFP, et al (2004) Strength training increases insulin-mediated glucose uptake, GLUT4 content, and insulin signaling in skeletal muscle in patients with type 2 diabetes. Diabetes 53: 294–305. 14747278 10.2337/diabetes.53.2.294

[33] Cornelissen VA, Fagard RH, Coeckelberghs E, Vanhees L (2011) Impact of resistance training on blood pressure and other cardiovascular risk factors. Hypertension 58: 950–958. 21896934 10.1161/HYPERTENSIONAHA.111.177071

