# Supplementary figures and images for: Muscle-Strengthening and Conditioning Activities and Risk of Type 2 Diabetes: A Prospective Study in Two Cohorts of US Women
Source: PLoS Med. 2014 Jan 14;11(1):e1001587. doi: 10.1371/journal.pmed.1001587 (PMC3891575; doi:10.1371/journal.pmed.1001587)

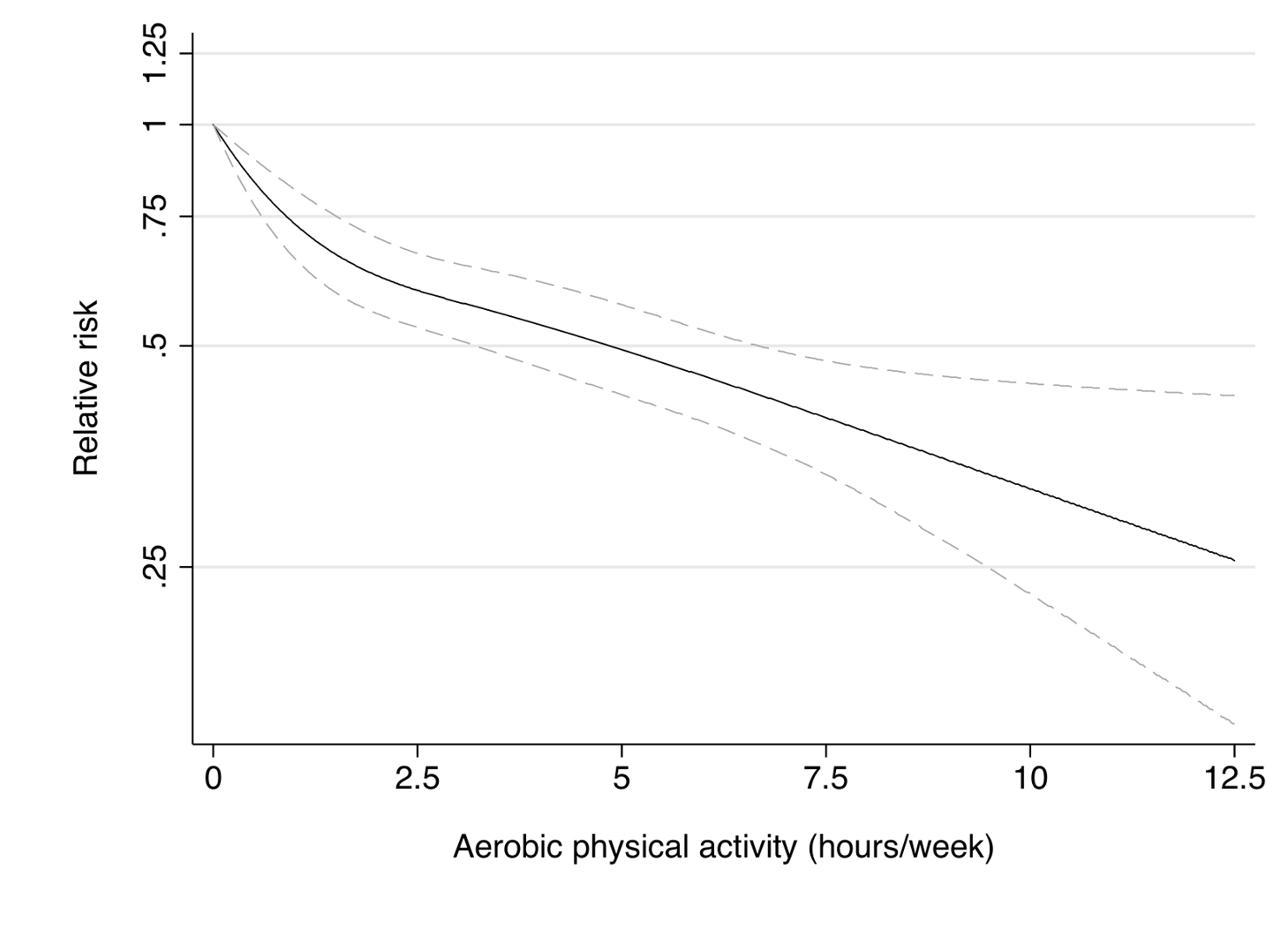

Supplement: Figure S1 — Dose-response relationship between aerobic physical activity (hours/week) and risk of type 2 diabetes in women from the Nurses' Health Study. Dotted lines are 95% CI for the trend obtained from restriced cubic spline regression (4 knots) truncated at 12.5 hours/week (≈99th percentile). Estimates were adjusted for age (months), smoking (never, past, or current), alcohol consumption (0, 1–5, >5 g/d), coffee intake (0, <1, 1–3, 3–5, >5 cups/day), race (white, non-white), family history of diabetes, post menopausal hormone use (never, past, current), intake of total energy, trans fat, polyunsaturated fat to saturated fat ratio, cereal fiber, wholegrain, and glycemic load (all dietary factors in quintiles), resistance exercise (0, 1–29, 30–59, 60–150, >150 min/week) and lower intensity muscular conditioning exercises (0, 1–29, 30–59, 60–150, >150 min/week). p<0.001 for non-linear response. (TIF) [file pmed.1001587.s001.tif]

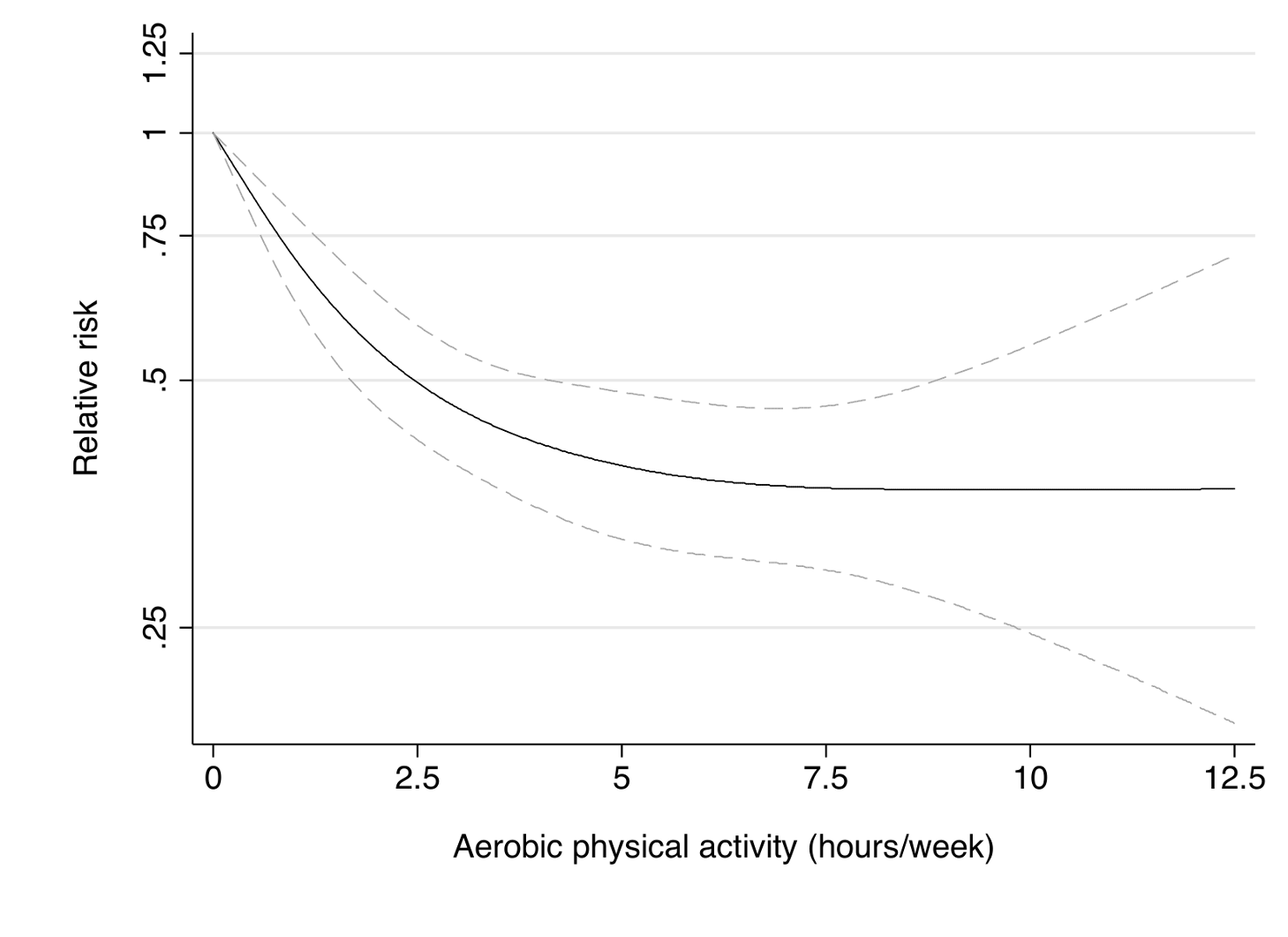

Supplement: Figure S2 — Dose-response relationship between aerobic physical activity (hours/week) and risk of type 2 diabetes in women from the Nurses' Health Study II. Dotted lines are 95% CI for the trend obtained from restriced cubic spline regression (4 knots) truncated at 12.5 hours/week (≈99th percentile). Estimates were adjusted for age (months), smoking (never, past, or current), alcohol consumption (0, 1–5, >5 g/d), coffee intake (0, <1, 1–3, 3–5, >5 cups/day), race (white, non-white), family history of diabetes, post menopausal hormone use (never, past, current), intake of total energy, trans fat, polyunsaturated fat to saturated fat ratio, cereal fiber, wholegrain, and glycemic load (all dietary factors in quintiles), oral contraceptive use (never, past, current), menopausal status (pre, post), resistance exercise (0, 1–29, 30–59, 60–150, >150 min/week) and lower intensity muscular conditioning exercises (0, 1–29, 30–59, 60–150, >150 min/week). p<0.001 for non-linear response. (TIF) [file pmed.1001587.s002.tif]
