# Supplementary material for: Muscle-Strengthening and Conditioning Activities and Risk of Type 2 Diabetes: A Prospective Study in Two Cohorts of US Women
Source: PLoS Med. 2014 Jan 14;11(1):e1001587. doi: 10.1371/journal.pmed.1001587 (PMC3891575; doi:10.1371/journal.pmed.1001587)
Supplement: Table S1 — Total muscle-strengthening activities and risk of type 2 diabetes in women from the Nurses' Health Study and Nurses' Health Study II using a 2-year lag between exposure and incidence of T2D. Data are relative risks (95% CI). *adjusted for age (months), smoking (never, past, or current), alcohol consumption (0, 1–5, >5 g/d), coffee intake (0, <1, 1–3, 3–5, >5 cups/day), race (white, non-white), family history of diabetes, post menopausal hormone use (never, past, current), intake of total energy, trans fat, polyunsaturated fat to saturated fat ratio, cereal fiber, wholegrain, and glycemic load (all dietary factors in quintiles), oral contraceptive use (only NHSII: never, past, current), menopausal status (only NHSII: pre, post), aerobic physical activity (categorized similar to muscle-strengthening activities). ** Combined using fixed effect pooling. (DOCX) [file pmed.1001587.s003.docx]

**Table S1.** Total muscle strengthening activities and risk of type 2 diabetes in women from the Nurses’ Health Study and Nurses’ Health Study II using a 2-year lag between exposure and incidence of T2D.

|  | **Minutes/week of muscle strengthening activity** | | | | |  |
| --- | --- | --- | --- | --- | --- | --- |
|  | None | 1 - 29 | 30 - 59 | 60-150 | >150 | p trend |
| **Nurses’ Health Study** |  |  |  |  |  |  |
| Cases/person years | 1,026/148,398 | 196/31,832 | 173/27,428 | 156/34,351 | 56/16,390 |  |
| Multivariable adjusted model* | 1.00 | 0.97 (0.82-1.13) | 1.08 (0.92-1.28) | 0.85 (0.71-1.00) | 0.74 (0.56-0.97) | 0.01 |
| **Nurses’ Health Study II** |  |  |  |  |  |  |
| Cases/person years | 652/125,144 | 138/42,259 | 101/29,323 | 106/43,346 | 57/25,769 |  |
| Multivariable adjusted model* | 1.00 | 0.74 (0.61-0.90) | 0.88 (0.71-1.10) | 0.71 (0.57-0.89) | 0.71 (0.54-0.95) | 0.007 |
|  |  |  |  |  |  |  |
| **Pooled results******** | 1.00 | 0.87 (0.77-0.98) | 1.01 (0.88-1.15) | 0.79 (0.69-0.91) | 0.72 (0.59-0.88) | <0.001 |

Data are relative risks (95% CI).

*adjusted for age (months), smoking (never, past, or current), alcohol consumption (0, 1-5, >5 g/d), coffee intake (0, <1, 1-3, 3-5, >5 cups/day), race (white, non-white), family history of diabetes, post menopausal hormone use (never, past, current), intake of total energy, trans fat, polyunsaturated fat to saturated fat ratio, cereal fiber, wholegrain, and glycemic load (all dietary factors in quintiles), oral contraceptive use (only NHSII: never, past, current), menopausal status (only NHSII: pre, post), aerobic physical activity (categorized similar to muscle-strengthening activities).

** Combined using fixed effect pooling.
