# Supplementary material for: Muscle-Strengthening and Conditioning Activities and Risk of Type 2 Diabetes: A Prospective Study in Two Cohorts of US Women
Source: PLoS Med. 2014 Jan 14;11(1):e1001587. doi: 10.1371/journal.pmed.1001587 (PMC3891575; doi:10.1371/journal.pmed.1001587)
Supplement: Table S2 — Muscle-strengthening and conditioning activities and risk of type 2 diabetes in women from the Nurses' Health Study (2000–2008) and Nurses' Health Study II (2001–2009) based on the simple updated activity level (recent activity level). All analyses were adjusted for age (months), smoking (never, past, or current), alcohol consumption (0, 1–5, >5 g/d), coffee intake (0, <1, 1–3, 3–5, >5 cups/day), race (white, non-white), family history of diabetes, post menopausal hormone use (never, past, current), intake of total energy, trans fat, polyunsaturated fat to saturated fat ratio, cereal fiber, wholegrain, and glycemic load (all dietary factors in quintiles), oral contraceptive use (only NHSII: never, past, current), menopausal status (only NHSII: pre, post), aerobic physical activity (categorized similar to muscle-strengthening activities). Estimates of associations for resistance exercise and lower intensity muscular conditioning exercises were mutually adjusted for each other. *Combined using fixed effect pooling. (DOCX) [file pmed.1001587.s004.docx]

**Table S2.** Muscle strengthening- and conditioning activities and risk of type 2 diabetes in women from the Nurses’ Health Study (2000-2008) and Nurses’ Health Study II (2001-2009) based on the simple updated activity level (recent activity level).

|  | **Minutes/week of activity** | | | | |  |  |
| --- | --- | --- | --- | --- | --- | --- | --- |
|  | None | 1 -29 | 30 -59 | 60-150 | >150 | p trend | RR per 60 min/week |
| **Nurses’ Health Study** |  |  |  |  |  |  |  |
| Total muscle strengthening-and conditioning activities | 1.00 | 0.85 (0.74-0.98) | 0.90 (0.77-1.04) | 0.78 (0.67-0.90) | 0.67 (0.54-0.83) | <0.001 | 0.92 (0.89-0.96) |
| Resistance exercise | 1.00 | 0.84 (0.70-1.01) | 0.74 (0.57-0.97) | 0.87 (0.72-1.05) | 0.72 (0.47-1.08) | 0.03 | 0.96 (0-90-1.01) |
| Lower intensity muscular conditioning exercises | 1.00 | 0.82 (0.70-0.96) | 0.93 (0.80-1.07) | 0.77 (0.64-0.92) | 0.59 (0.37-0.94) | <0.001 | 0.92 (0.86-0.97) |
|  |  |  |  |  |  |  |  |
| **Nurses’ Health Study II** |  |  |  |  |  |  |  |
| Total muscle strengthening-and conditioning activities | 1.00 | 0.74 (0.62-0.88) | 0.81 (0.66-0.99) | 0.62 (0.51-0.75) | 0.61 (0.47-0.79) | <0.001 | 0.91 (0.87-0.96) |
| Resistance exercise | 1.00 | 0.70 (0.57-0.87) | 0.63 (0.44-0.90) | 0.74 (0.60-0.91) | 0.71 (0.46-1.09) | 0.02 | 0.93 (0.87-0.99) |
| Lower intensity muscular conditioning exercises | 1.00 | 0.86 (0.72-1.04) | 0.84 (0.69-1.01) | 0.84 (0.65-1.07) | 0.58 (0.29-1.17) | 0.03 | 0.90 (0.83-0.99) |
|  |  |  |  |  |  |  |  |
| **Pooled results*** |  |  |  |  |  |  |  |
| Total muscle strengthening-and conditioning activities | 1.00 | 0.80 (0.72-0.90) | 0.86 (0.76-0.98) | 0.72 (0.64-0.81) | 0.64 (0.54-0.76) | <0.001 | 0.92 (0.89-0.95) |
| Resistance exercise | 1.00 | 0.78 (0.68-0.89) | 0.70 (0.56-0.87) | 0.81 (0.71-0.93) | 0.71 (0.53-0.96) | 0.002 | 0.94 (0.90-0.98) |
| Lower intensity muscular conditioning exercises | 1.00 | 0.84 (0.74-0.95) | 0.89 (0.79-1.00) | 0.79 (0.68-0.92) | 0.58 (0.40-0.86) | <0.001 | 0.91 (0.87-0.96) |

Data are relative risk (95% CI). All analyses were adjusted for age (months), smoking (never, past, or current), alcohol consumption (0, 1-5, >5 g/d), coffee intake (0, <1, 1-3, 3-5, >5 cups/day), race (white, non-white), family history of diabetes, post menopausal hormone use (never, past, current), intake of total energy, trans fat, polyunsaturated fat to saturated fat ratio, cereal fiber, wholegrain, and glycemic load (all dietary factors in quintiles), oral contraceptive use (only NHSII: never, past, current), menopausal status (only NHSII: pre, post), aerobic physical activity (categorized similar to muscle-strengthening activities). Estimates of associations for resistance exercise and lower intensity muscular conditioning exercises were mutually adjusted for each other.* Combined using fixed effect pooling
