# Supplementary material for: Muscle-Strengthening and Conditioning Activities and Risk of Type 2 Diabetes: A Prospective Study in Two Cohorts of US Women
Source: PLoS Med. 2014 Jan 14;11(1):e1001587. doi: 10.1371/journal.pmed.1001587 (PMC3891575; doi:10.1371/journal.pmed.1001587)
Supplement: Table S3 — Muscle-strengthening activities and risk of type 2 diabetes stratified by age (<65, ≥65 years, NHS only), family history of T2D, diet quality, race (white, non-white), and aerobic physical activity (quintiles). Data are relative risks (95% CI). Multivariable model included age (months), smoking (never, past, or current), alcohol consumption (0, 1–5, >5 g/d), coffee intake (0, <1, 1–3, 3–5, >5 cups/day), race (white, non-white), family history of diabetes, post menopausal hormone use (never, past, current), intake of total energy, trans fat, polyunsaturated fat to saturated fat ratio, cereal fiber, wholegrain, and glycemic load (all dietary factors in quintiles), oral contraceptive use (only NHSII: never, past, current), menopausal status (only NHSII: pre, post), aerobic physical activity (none, 1–29, 30–59, 60–150, >150 min/week). *Only adjusted for age and aerobic physical activity (none, 1–29, 30–59, 60–150, >150 min/week) due to the low number of cases in some groups. (DOCX) [file pmed.1001587.s005.docx]

**Table S3.** Muscle strengthening activities and risk of type 2 diabetes stratified by age (<65, ≥65 years, NHS only), family history of T2D, diet quality, race (white, non-white), and aerobic physical activity (quintiles).

|  | **No** | **Person-years** | **Cases** | **RR per 60 min/week** |
| --- | --- | --- | --- | --- |
| **Nurses’ Health Study** |  |  |  |  |
| **Age (years)** (Nurses’ Health Study only) |  |  |  |  |
| <65 | 25,913 | 131,901 | 808 | 0.90 (0.83-0.97) |
| ≥65 | 37,832 | 213,851 | 1,350 | 0.91 (0.86-0.96) |
| **Family history of type 2 diabetes** |  |  |  |  |
| Negative | 38,224 | 256,940 | 1,235 | 0.89 (0.83-0.94) |
| Positive | 13,418 | 88,813 | 923 | 0.93 (0.87-0.99) |
| **Dietary index score** |  |  |  |  |
| <Median | 29,403 | 172,724 | 1,277 | 0.91 (0.85-0.98) |
| >Median | 29,157 | 173,028 | 881 | 0.90 (0.84-0.96) |
| **Race*** |  |  |  |  |
| White | 50,502 | 338,354 | 2,084 | 0.87 (0.83-0.92) |
| Non-white | 1,140 | 7,398 | 74 | 0.95 (0.77-1.17) |
| **Aerobic physical activity*** |  |  |  |  |
| Q1 | 13,693 | 67,656 | 655 | 0.79 (0.69-0.90) |
| Q2 | 15,456 | 69,998 | 536 | 0.92 (0.82-1.03) |
| Q3 | 15,101 | 69,111 | 414 | 0.94 (0.85-1.04) |
| Q4 | 14,418 | 69,442 | 342 | 0.88 (0.79-0.97) |
| Q5 | 12,085 | 69,545 | 211 | 0.88 (0.80-0.96) |
|  |  |  |  |  |
| **Nurses’ Health Study II** |  |  |  |  |
| **Family history of type 2 diabetes** |  |  |  |  |
| Negative | 30,965 | 235,521 | 495 | 0.91 (0.83-1.00) |
| Positive | 16,709 | 124,867 | 838 | 0.93 (0.87-0.99) |
|  |  |  |  |  |
| **Dietary index score** |  |  |  |  |
| <Median | 27,090 | 179,978 | 788 | 0.91 (0.84-0.98) |
| >Median | 26,940 | 180,140 | 545 | 0.95 (0.88-1.01) |
| **Race*** |  |  |  |  |
| White | 46,317 | 350,119 | 1,266 | 0.90 (0.85-0.95) |
| Non-white | 1,357 | 9,999 | 67 | 1.01 (0.90-1.14) |
| **Aerobic physical activity*** |  |  |  |  |
| Q1 | 12,753 | 72,581 | 511 | 0.74 (0.60-0.89) |
| Q2 | 14,386 | 70,709 | 307 | 0.86 (0.72-1.03) |
| Q3 | 14,776 | 72,352 | 244 | 0.92 (0.81-1.04) |
| Q4 | 13,944 | 72,287 | 158 | 0.88 (0.77-1.00) |
| Q5 | 11,580 | 72,190 | 113 | 1.00 (0.94-1.07) |

Data are relative risks (95% CI). Multivariable model included age (months), smoking (never, past, or current), alcohol consumption (0, 1-5, >5 g/d), coffee intake (0, <1, 1-3, 3-5, >5 cups/day), race (white, non-white), family history of diabetes, post menopausal hormone use (never, past, current), intake of total energy, trans fat, polyunsaturated fat to saturated fat ratio, cereal fiber, wholegrain, and glycemic load (all dietary factors in quintiles), oral contraceptive use (only NHSII: never, past, current), menopausal status (only NHSII: pre, post), aerobic physical activity (none, 1-29, 30-59, 60-150, >150 min/week). *Only adjusted for age and aerobic physical activity (none, 1-29, 30-59, 60-150, >150 min/week) due to the low number of cases in some groups.
